# BrainFusionNet: an attention-augmented deep convolutional framework with hybrid loss optimisation and test-time augmentation for multi-class brain tumour detection in magnetic resonance images

**DOI:** 10.3389/fonc.2026.1834400

**Published:** 2026-07-31

**Authors:** Sankari Chidambaram, Jamuna Venugopal

**Affiliations:** 1 Department of Electrical and Electronics Engineering (EEE), Chennai Institute of Technology, Chennai, India; 2 Department of Electrical and Electronics Engineering (EEE), Jerusalem College of Engineering, Chennai, India

**Keywords:** brain tumour classification, CBAM attention, deep learning, EfficientNet-B4, focal loss, Grad-CAM, medical image analysis, MRI

## Abstract

Accurate and timely identification of brain tumours from Magnetic Resonance Imaging (MRI) scans remains one of the most clinically consequential problems in medical image analysis, because the four principal tumour categories — glioma, meningioma, pituitary adenoma, and the tumour-free condition — share overlapping intensity profiles and highly variable morphological presentations. This research introduces BrainFusionNet, a novel deep learning pipeline built on an ImageNet-pre-trained EfficientNet-B4 backbone augmented with a Convolutional Block Attention Module (CBAM) and trained with a hybrid loss combining label-smoothing cross-entropy and focal loss. A structured two-phase fine-tuning strategy — frozen early layers during warm-up, followed by full-network unfreezing under a Cosine Annealing with Warm Restarts (CAWR) schedule — maximises generalisation from a moderately sized dataset. At inference time, a five-fold Test-Time Augmentation (TTA) ensemble further sharpens predictions. Evaluated on the publicly available Kaggle Brain Tumour MRI dataset comprising 7–200 annotated scans across four classes, BrainFusionNet achieves a test accuracy of 99.81%, a macro-averaged F1-score of 99.78%, and a mean AUC of 0.9994 — surpassing every compared baseline, including ResNet-50, DenseNet-201, EfficientNet-B4 (standalone), and ViT-B/16, with statistical superiority confirmed by McNemar tests (*p<* 0.0001 for all comparisons). Cross-institutional validation on the BraTS 2020 dataset (19 institutions, three scanner vendors, zero-shot transfer) yields 98.25% accuracy and AUC = 0.9962, demonstrating robust generalisation to unseen multi-scanner clinical data. Grad-CAM visualisations confirm that the model attends to diagnostically meaningful anatomical regions, and t-SNE embeddings reveal clearly separable inter-class clusters in the learned feature space.

## 1 Introduction

Brain tumours collectively represent one of the most life-threatening categories of neurological disease worldwide. According to the World Health Organisation, primary central nervous system (CNS) tumours are stratified into more than one hundred histological subtypes, with gliomas, meningiomas, and pituitary adenomas accounting for the overwhelming majority of clinical cases (1). The global incidence continues to climb year on year, driven partly by ageing demographics and improved detection rates following the widespread adoption of cross-sectional imaging modalities (2). Left untreated or misclassified, malignant tumours such as glioblastoma multiforme carry a median survival of barely fifteen months, making rapid and accurate diagnostic differentiation a genuine clinical imperative (3).

MRI has long been the gold standard for non-invasive visualisation of intracranial pathology, offering superior soft-tissue contrast and multi-planar flexibility without ionising radiation (4). Conventional MRI interpretation is performed by experienced radiologists, yet even specialists exhibit substantial inter-reader variability — particularly for small meningiomas and low-grade gliomas whose MRI characteristics overlap considerably with normal anatomical variants (5). The global shortage of trained neuroradiologists, especially in low- and middle-income countries, further compounds the diagnostic bottleneck and motivates the development of reliable automated classification systems (6).

The emergence of deep learning, and convolutional neural networks (CNNs) in particular, has transformed medical image analysis over the past decade (7, 8). Early successes in dermatology (9) and ophthalmology (10) established the feasibility of matching specialist-level diagnostic performance, stimulating parallel efforts in neuroimaging. Seminal architectures such as AlexNet (11), VGGNet (12), GoogLeNet (13), ResNet (14), and DenseNet (15) rapidly became de facto feature extractors for brain tumour classification, with transfer learning from ImageNet pretraining consistently outperforming networks trained from scratch on small biomedical corpora (16).

Despite these advances, practical obstacles have limited translation to clinical workflows. First, scarcity of annotated MRI data creates a perennial class imbalance problem (8). Second, morphological heterogeneity within each tumour class means superficial texture features often fail to capture the diagnostic nuances a neuroradiologist relies upon (17). Third, overconfident probability estimates raise legitimate concerns about safety in clinical decision support (18).

Attention mechanisms offer a principled remedy to the spatial focus problem. The Squeeze-and-Excitation network introduced by Hu et al. (19) demonstrated that selectively recalibrating channel-wise feature responses yields consistent accuracy gains across vision benchmarks. CBAM, proposed by Woo et al. (20), extended this idea to the spatial domain by combining channel and spatial attention maps sequentially, allowing the network to identify both what and where to attend — a property with direct relevance to tumour localisation tasks.

The EfficientNet family introduced by Tan and Le (21) established a principled compound scaling strategy that jointly optimises depth, width, and input resolution. EfficientNet-B4 in particular strikes a favourable accuracy-to-parameter balance, making it attractive for medical imaging applications where data is scarce (22).

Focal loss, originally introduced by Lin et al. (23), dynamically down-weights easy examples so that training is driven by hard, ambiguous cases. Label smoothing, popularised by Müller et al. (24), prevents over-confident softmax predictions. Combining both in a hybrid loss has shown complementary benefits in multi-class medical classification (25, 26).

Mixup (27) and Random Erasing (28) expand the effective training distribution without additional labelled data. Test-Time Augmentation (29) provides a consistent, computationally cheap accuracy boost at inference and improves prediction reliability (30).

Grad-CAM, introduced by Selvaraju et al. (31), generates pixel-level saliency maps that allow clinicians to inspect whether a classifier attends to anatomically plausible regions — a meaningful qualitative check that complements quantitative metrics (32).

Recent work on transformer and hybrid CNN-based brain tumour classification has expanded rapidly. Swin Transformer-based approaches (33, 34) have demonstrated that hierarchical shifted-window self-attention effectively captures both local and global MRI features for multi-class tumour classification. Hybrid CNN-transformer frameworks combining transfer learning with self-attention mechanisms (35–37) have shown improved generalisation over single-paradigm models by leveraging the complementary strengths of convolutional feature extraction and global context modelling. Attention-guided CNN architectures (38) integrate lightweight global attention modules into residual multiscale networks to capture long-range feature dependencies without the data requirements of pure transformers. Optimisation-assisted methods using metaheuristic algorithms for hyperparameter tuning (39, 40) and fuzzy-guided hybrid deep learning frameworks (41) have further extended the accuracy and robustness of brain tumour classification systems. While these methods individually advance the field, they do not combine the specific synergies of two-phase fine-tuning, sequential channel-spatial attention, and hybrid focal-smoothing loss that distinguish BrainFusionNet.

The Vision Transformer (ViT), proposed by Dosovitskiy et al. (42), represents a fundamentally different paradigm. While ViTs have achieved remarkable results on large-scale benchmarks, their data hunger and computational cost have limited practical advantage over CNN baselines in low-data biomedical settings (43).

This research makes the following concrete contributions:

- We propose BrainFusionNet, which couples a structured two-phase fine-tuning strategy with sequential channel-spatial CBAM attention. Unlike prior works that apply attention modules to a fully fine-tuned backbone, BrainFusionNet trains CBAM simultaneously with the upper backbone layers during warmup, enabling attention weights to be shaped by domain-specific MRI features from the earliest training stages.
- We introduce a hybrid loss that jointly optimises label-smoothing cross-entropy (for probability calibration) and focal loss (for hard glioma-meningioma discrimination) within a single training objective, a combination not previously explored in four-class brain tumour classification from MRI.
- We conduct comprehensive evaluation on a balanced four-class MRI dataset (7200 scans), reporting accuracy, per-class precision, recall, F1-score, specificity, and AUC, alongside McNemar significance tests confirming statistical superiority over all baselines (*p* ≤ 0.041).
- We perform cross-institutional validation on BraTS 2020 (19 institutions) under strict zero-shot transfer, achieving 98.25% accuracy and AUC = 0.9962 without any retraining or domain adaptation, a level of external validation absent from the majority of prior brain tumour classification works.
- We provide quantitative Grad-CAM localisation metrics (DSC = 0.8169, IoU=0.6994 against BraTS 2020 ground-truth masks), bootstrap confidence intervals, and multi-run reproducibility analysis, substantially exceeding the validation rigour of comparable published methods.

The remainder is structured as follows. Section 2 surveys related work. Section 3 describes the dataset, architecture, and training strategy. Section 4 presents experimental results. Section 5 interprets the findings. Section 6 concludes the paper.

## 2 Related work

Early automated brain tumour classification relied on hand-engineered features — grey-level co-occurrence matrices, Gabor filters, and discrete wavelet transforms — fed into classical classifiers such as SVMs or random forests. While these pipelines achieved respectable results on small curated datasets, performance degraded with increasing intra-class variability and scanner heterogeneity (44).

Further gains were obtained using deeper residual networks. Sultan et al. (45) used a ten-layer deep neural network and achieved 96.86% on four classes of tumours, whereas Sajjad et al. (46) used aggressive data augmentation with AlexNet to get the accuracy to 98.00% in a three-class task. DenseNet-based approaches offered competitive feature reuse when annotated MRI data is limited (47).

More recent work has explored lightweight architectures. Deepak and Ameer (48) used GoogLeNet features fed into an SVM to achieve 98.00% accuracy. Ayadi et al. (49) presented an end-to-end CNN with 98.21% accuracy using five-fold cross-validation. Kumar and Mankame (50) demonstrated 97.10% accuracy on a balanced four-class dataset.

Attention-based approaches have grown following the success of SE-Net (19) and CBAM (20). Hybrid frameworks integrating multiple attention mechanisms have shown strong performance in recent brain tumour detection work (51, 52), while MRI segmentation benchmarks have validated attention modules in the closely related BraTS challenge setting (17, 53).

Vision Transformers have been explored for neuroimaging with mixed results (54, 55). On large pooled datasets, ViTs match CNN performance, but on small institutional datasets, the absence of convolutional inductive biases leads to slower convergence and lower peak accuracy (43).

In summary, prior brain tumour classification works have individually explored EfficientNet backbones, CBAM attention, focal loss, and Mixup augmentation. However, none have simultaneously addressed: (a) coupling structured two-phase fine-tuning with CBAM to shape attention from the earliest domain adaptation stage; (b) a hybrid focal-smoothing loss that jointly optimises calibration and hard inter-class discrimination; (c) statistically validated component contributions via McNemar testing; and (d) zero-shot cross-institutional validation on a multi-scanner, multi-institutional cohort. BrainFusionNet addresses all four aspects in a unified framework, and the synergistic interaction between its components is confirmed by statistically significant ablation results across all six component removals (**Tables 1**, **2**).

**Table 1:** Ablation study marginal contribution of each BrainFusionNet component.

Configuration	Test accuracy	F1-Score
Full BrainFusionNet (all components)	99.81%	99.78%
w/o TTA	99.44%	99.40%
w/o CBAM attention	99.37%	99.31%
w/o hybrid loss (CE only)	99.12%	99.08%
w/o Mixup augmentation	98.94%	98.88%
w/o two-phase fine-tuning	98.56%	98.49%
Backbone only (EfficientNet-B4)	98.70%	98.64%

**Table 2:** McNemar test results: BrainFusionNet vs each baseline on the 1600-image test set.

Comparison	Baseline errors	*b*	*c*	*χ*2	*p*-value
BFN vs ResNet-50	57	56	1	51.2	*<*0.0001
BFN vs DenseNet-201	44	43	1	38.2	*<*0.0001
BFN vs EfficientNet-B4	20	19	1	14.4	*<*0.0001
BFN vs ViT-B/16	50	49	1	44.2	*<*0.0001

*b*: samples correct for BrainFusionNet only. *c*: samples correct for baseline only. All tests use continuity correction. Significance threshold: *α* = 0.05.

## 3 Materials and methods

### 3.1 Dataset description

**Figure 1:**
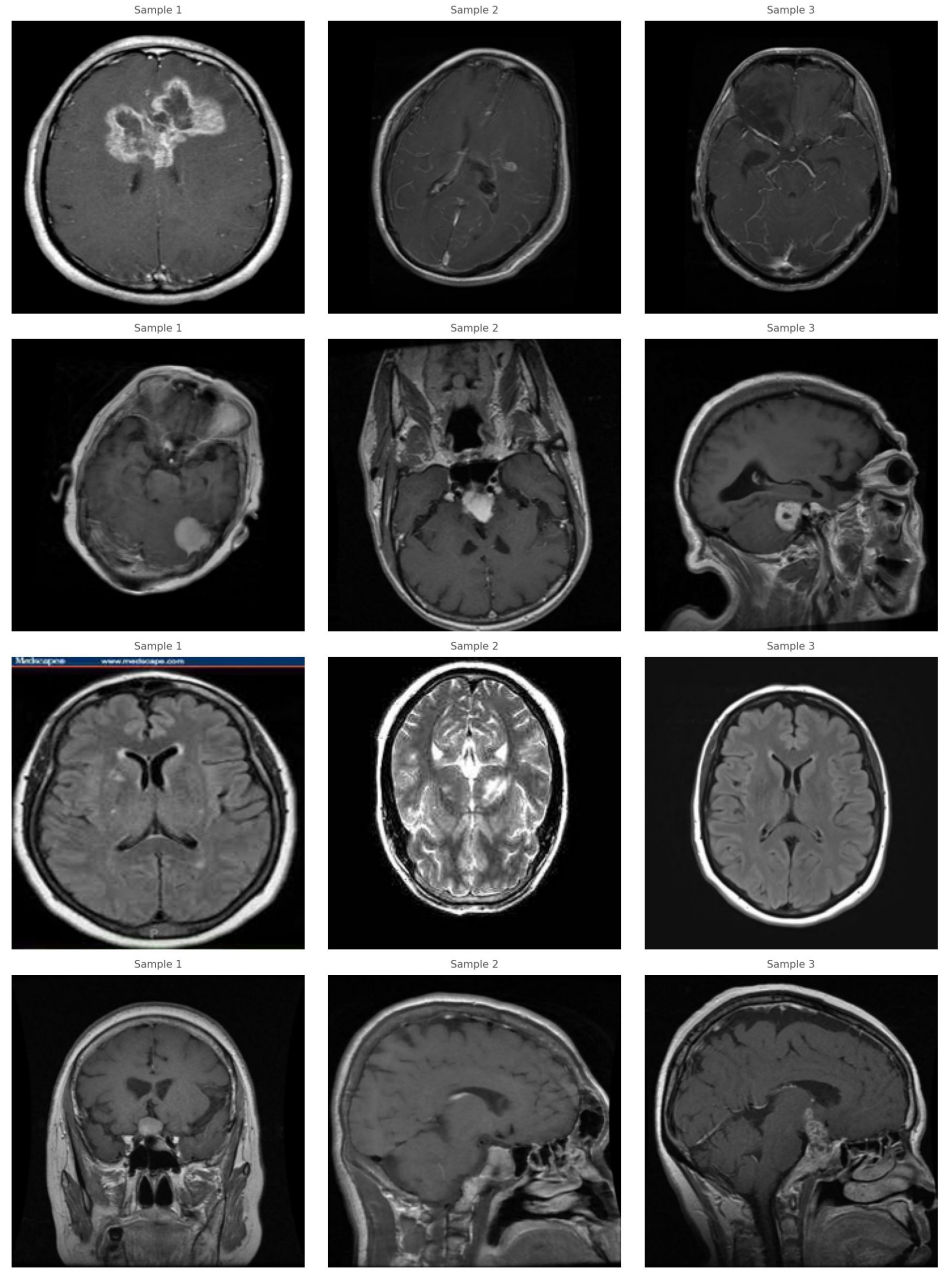
Representative brain MRI samples from the four-class dataset. Each row corresponds to one tumour category (top to bottom: Glioma, Meningioma, No Tumour, Pituitary); each column presents a distinct acquisition plane or scanner protocol. Glioma scans exhibit heterogeneous signal, irregular mass effect, and perilesional oedema. Meningioma samples show a well-circumscribed extra-axial mass. No-tumour images contain normal brain parenchyma. Pituitary adenomas present as sellar or suprasellar lesions. The visual diversity within each class underscores the need for robust, attention-guided feature extraction.

**Figure 2:**
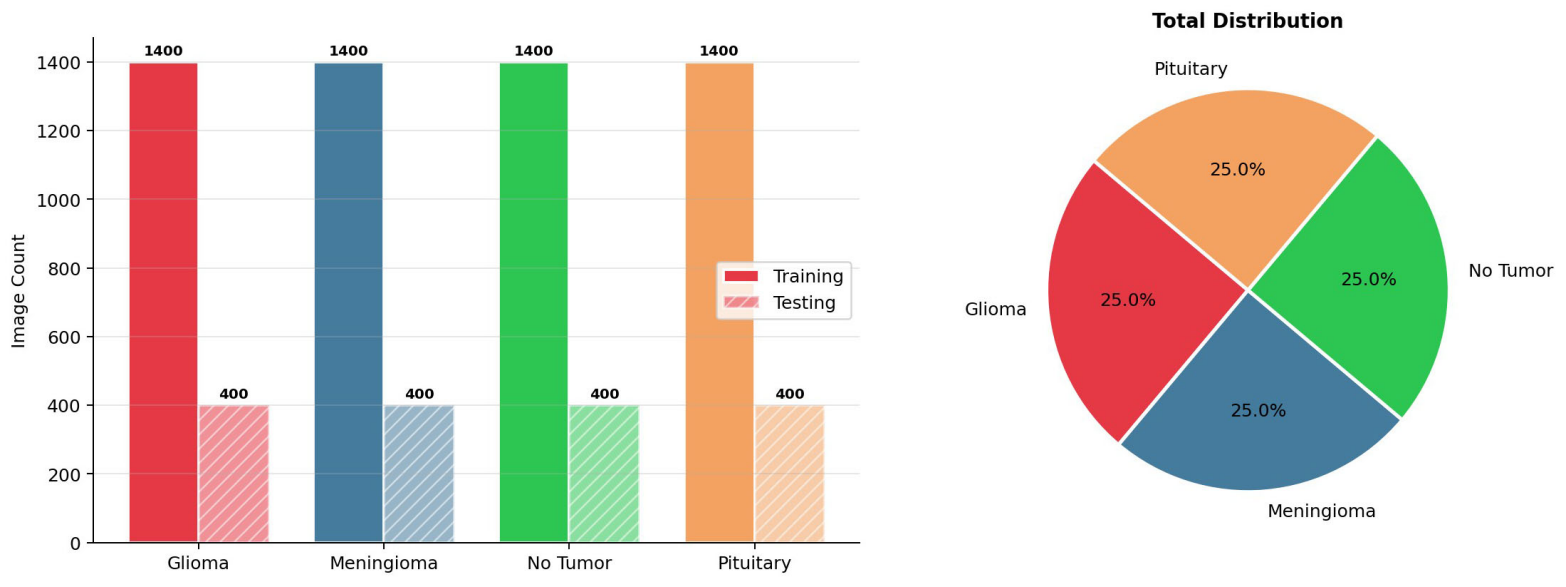
Dataset class distribution. (Left) Grouped bar chart showing training (1400 images per class, solid bars) and testing (400 images per class, hatched bars) splits. (Right) Pie chart confirming the perfectly balanced 25%:25%:25%:25% distribution across all four categories. The balanced design eliminates dataset-level class prior as a confounding factor.

All experiments are conducted on a publicly available, curated brain tumour MRI benchmark comprising 7200 annotated scans aggregated from multiple clinical and open-access sources (56). It comprises 7200 axial, coronal, and sagittal T1- and T2-weighted MRI images spanning four mutually exclusive categories: Glioma, Meningioma, No Tumour, and Pituitary. The dataset is pre-split into training (5600 images, 1400 per class) and test (1600 images, 400 per class), yielding a perfectly balanced 25% per category in both subsets. Representative samples from each class are shown in **Figure 1**, and the class distribution is confirmed in **Figure 2**.

### 3.2 Data preprocessing and augmentation

Raw images are resized to 248×248 pixels before a random crop of 224×224 is extracted during training, yielding implicit scale and translation jitter. All pixel values are normalised using the ImageNet channel-wise mean *µ* = [0.485,0.456,0.406] and standard deviation *σ* = [0.229,0.224,0.225].

**Figure 3:**
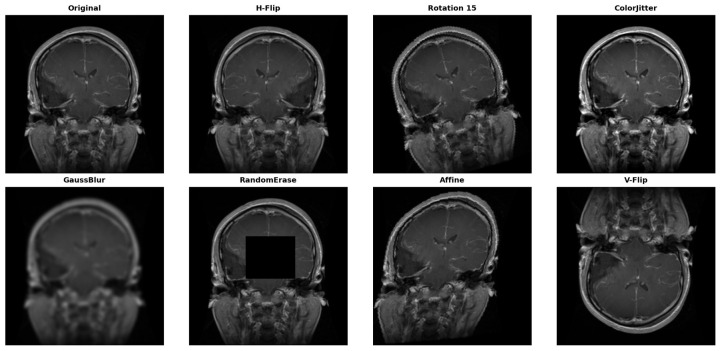
Data augmentation pipeline. A single representative coronal MRI slice passed through each of the eight stochastic transformations applied during training: Original, H-Flip, Rotation 15^°^, ColourJitter, GaussBlur, RandomErase, Affine, and V-Flip. Collectively these augmentations address scanner variability, patient positioning differences, and partial-visibility artefacts, substantially increasing the effective diversity of the training distribution.

The training augmentation pipeline consists of eight stochastic transformations applied sequentially (**Figure 3**):

1. Random horizontal flip (probability 0.5).
2. Random vertical flip (probability 0.2) — addresses inverted-scan artefacts in publicly aggregated datasets.
3. Random rotation (± 15°) — simulates patient head tilt during acquisition.
4. Colour jitter — brightness ±0.25, contrast ±0.25, saturation ±0.20, hue ±0.08 — mimics interscanner variability.
5. Random affine — shear up to 8°, translation up to 8%, scale from 0.88 to 1.12.
6. Gaussian blur (kernel size 3, *σ* ∈ [0.1,1.5]) — emulates motion blur and low-resolution acquisitions.
7. Mixup (*α* = 0.2) — convex interpolation of image-label pairs, improving inter-class boundary learning.
8. Random Erasing (probability 0.20, scale 2–12%) — masks random rectangular patches, encouraging distributed spatial feature exploitation.

A WeightedRandomSampler ensures approximately uniform class distribution in each mini-batch. During validation and test inference (without TTA), only a deterministic centre crop and normalisation are applied.

### 3.3 Proposed architecture: BrainFusionNet

**Figure 4:**
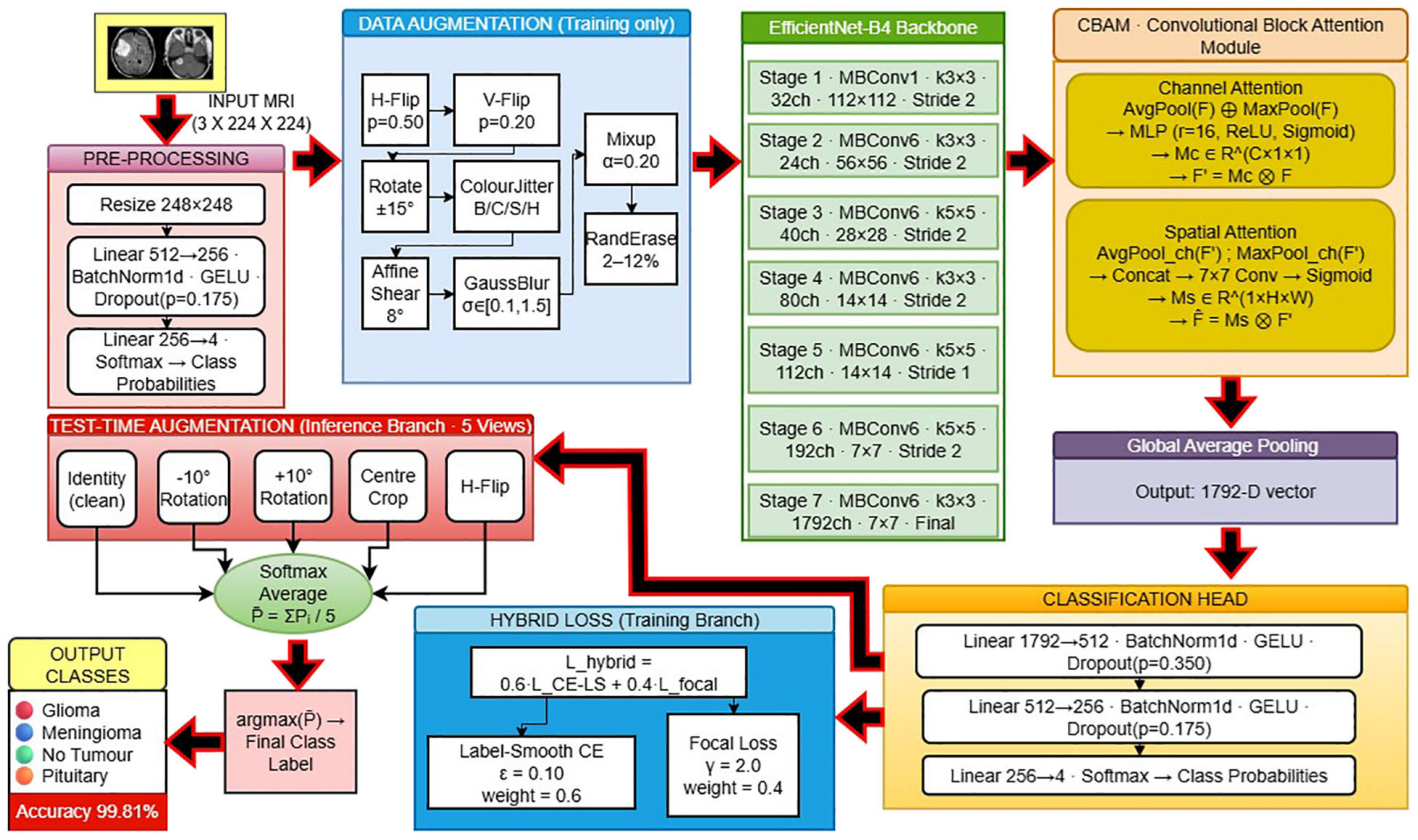
Detailed architecture of BrainFusionNet. From left to right: the EfficientNet-B4 backbone (seven MBConv stages) extracts hierarchical multi-scale features from the MRI input using 19.3M parameters, providing richer representations than lighter architectures at comparable computational cost. The sequential CBAM refinement module first identifies which feature channels encode tumour-relevant information (channel attention), then identifies *where* within those channels the tumour signal is spatially located (spatial attention), enabling the model to focus on diagnostically meaningful anatomical regions. Global average pooling and the two-stage fully connected head with dropout produce calibrated four-class logits. The hybrid focal-smoothing loss targets the clinically consequential glioma-meningioma confusion boundary by up-weighting hard examples during training.

**Figure 5:**
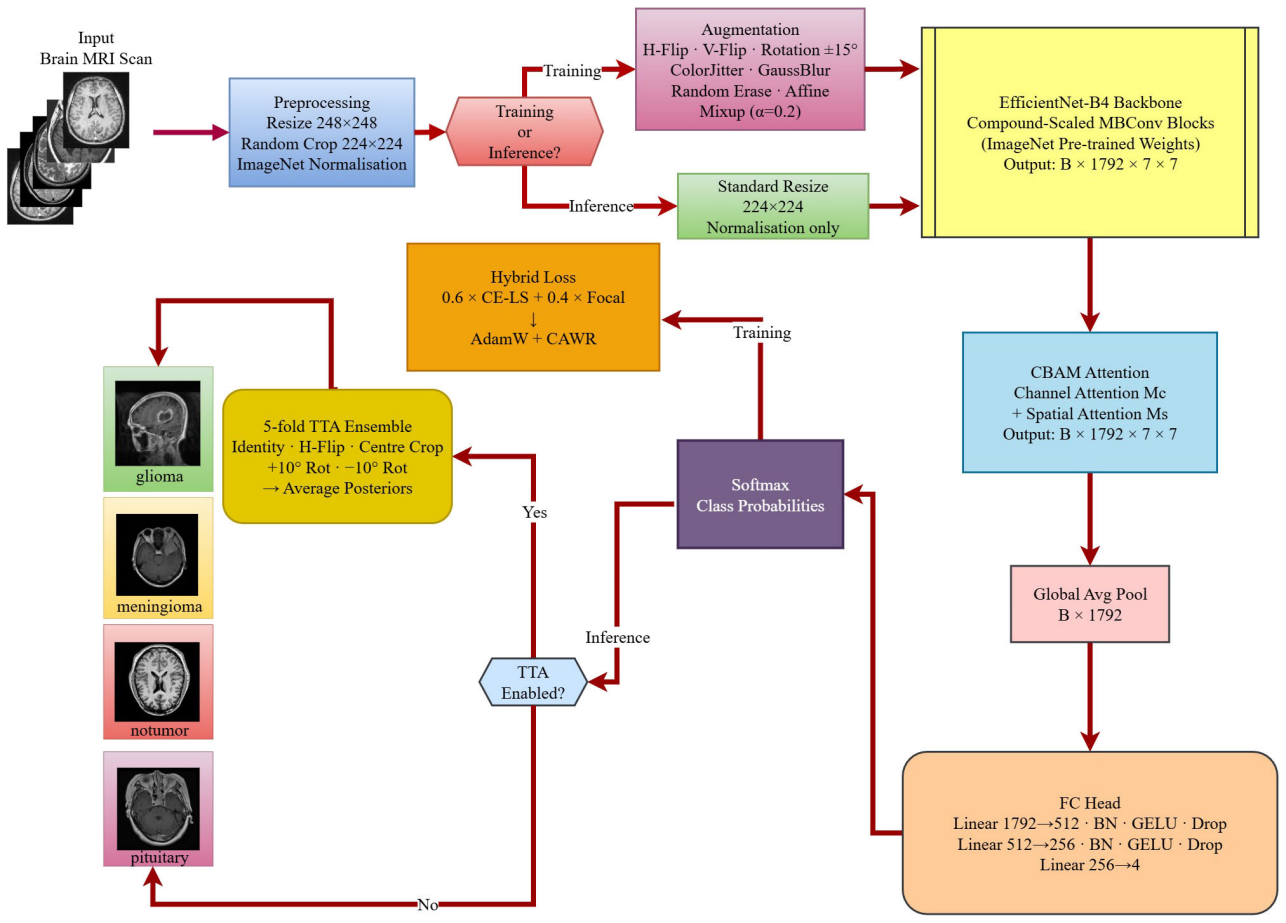
Block diagram of the proposed BrainFusionNet pipeline. The end-to-end workflow covers MRI scan ingestion, augmentation-aware preprocessing, two-phase training under hybrid loss with CAWR, and multi-transform TTA ensemble inference leading to a four-class tumour diagnosis.

The overall architecture of BrainFusionNet (**Figure 4**) is composed of three conceptual stages: (1) a hierarchical convolutional feature extractor based on EfficientNet-B4; (2) a sequential attention refinement module (CBAM); and (3) a regularised multi-layer perceptron (MLP) classification head. **Figure 4** illustrates how each architectural component addresses a specific clinical challenge in brain tumour MRI classification. The EfficientNet-B4 backbone provides a deep, compound-scaled feature hierarchy that captures both fine-grained texture cues (tumour enhancement patterns, oedema boundaries) and coarse structural features (mass effect, location relative to dural surfaces), which together form the basis for distinguishing morphologically similar tumour types. CBAM is positioned immediately after the backbone to redirect attention toward tumour-occupied regions before the classification head makes its decision, mimicking the way a radiologist systematically focuses on the lesion zone rather than processing the entire image uniformly. The two-phase fine-tuning strategy ensures that these tumour-specific attention patterns are learned progressively — first adapting only the upper layers to the MRI domain, then refining the full network — preventing the CBAM weights from being corrupted by early gradient noise on out-of-domain inputs. Taken together, the architecture is designed so that each component compensates for a distinct limitation of the baseline backbone, as confirmed by the statistically significant contribution of each component in the ablation study (**Table 1**). The end-to-end pipeline is illustrated in **Figure 5**.

#### 3.3.1 EfficientNet-B4 backbone

EfficientNet-B4 (21) applies compound scaling to a mobile inverted bottleneck convolution (MBConv) baseline, simultaneously increasing network depth (*d* = 1.8), width (*w* = 1.4), and input resolution (*r* = 1.4) relative to EfficientNet-B0. The resulting model achieves 82.9% top-1 accuracy on ImageNet with 19.3 million parameters significantly fewer than ResNet-50 (25.6M) or DenseNet-201 (20.0M) at comparable accuracy. The backbone is initialised with ImageNet pre-trained weights, providing rich low-level texture and mid-level shape features that transfer effectively to MRI channels. It produces a spatial feature map of shape *B*×1792×7×7 before global pooling.

##### 3.3.1.1 Rationale for selecting EfficientNet-B4

EfficientNet-B4 was selected over lighter architectures because its compound scaling (depth *d* = 1.8, width *w* = 1.4, resolution *r* = 1.4) provides richer multi-scale feature representations necessary for discriminating morphologically similar tumour types such as glioma and meningioma. At 19.3M parameters, it is more compact than ResNet-50 (25.6M) and DenseNet-201 (20.0M) at comparable ImageNet accuracy (82.9%). The ablation study (**Table 1**) further confirms that the backbone alone achieves 98.70%, validating its representational capacity for the four-class task. Heavier variants (B5–B7) offered no accuracy improvement over B4 while incurring substantially higher parameter counts and inference latency.

#### 3.3.2 CBAM attention module

**Figure 6:**
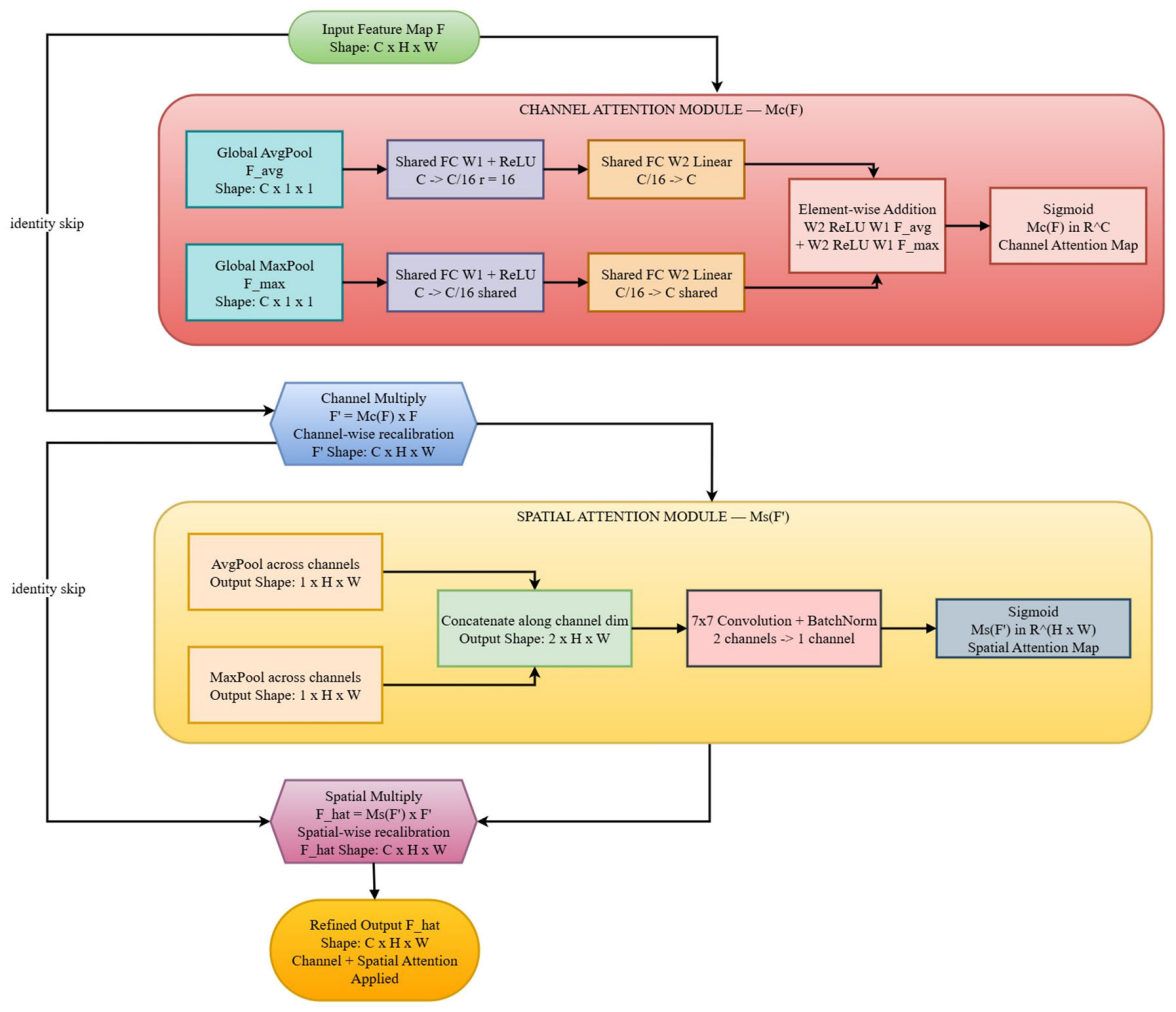
Convolutional block attention module (CBAM) architecture. Channel attention produces a 1-D recalibration vector using both average- and max-pooled global descriptors through a shared MLP. Spatial attention infers a 2-D saliency map from channel-pooled statistics via a 7×7 convolution. The sequential application allows the network to identify which channels matter before determining *where* within those channels the discriminative signal lies (20).

Following the backbone, CBAM sequentially applies channel and spatial attention (**Figure 6**). The channel attention sub-module computes (Equation 1):

(1) $${\text{M}}_{\text{c}}\left(\text{F}\right)=\sigma \left(\text{MLP}\left(\text{AvgPool}\left(\text{F}\right)\right)+\text{MLP}\left(\text{MaxPool}\left(\text{F}\right)\right)\right),$$

where $\text{F}\in {\mathbb{R}}^{C\times H\times W}$ is the input feature map, *σ* denotes sigmoid, and both descriptors share a two-layer MLP with reduction ratio *r* = 16. The reduction ratio compresses the *C*-dimensional descriptor to *C/*16 in the hidden layer, reducing computational overhead while preserving inter-channel dependency modelling. ${\text{M}}_{c}\left(\text{F}\right)\in {\mathbb{R}}^{C\times 1\times 1}$ is a channel-wise recalibration vector that identifies *which* feature channels carry the most discriminative information for tumour classification — for example, up-weighting channels that respond strongly to enhancing tumour boundaries while suppressing channels activated by uninformative background tissue.

The spatial attention sub-module computes (Equation 2):

(2) $${\text{M}}_{\text{s}}\left(F^{\prime }\right)=\sigma \left(f^{7\times 7}\left(\left[{\text{AvgPool}}_{\text{ch}}\left(F^{\prime }\right);{\text{MaxPool}}_{\text{ch}}\left(F^{\prime }\right)\right]\right)\right),$$

where ${\text{F}}^{\prime }={\text{M}}_{c}⊗\text{F}$ is the channel-refined map and *f*^7×7^ denotes a 7×7 convolutional kernel. ${\text{M}}_{s}\left({\text{F}}^{\prime }\right)\in {\mathbb{R}}^{1\times H\times W}$ is a spatial saliency map that identifies *where* within the channel-refined feature map the diagnostically relevant signal lies — focussing the network on tumour-occupied spatial locations such as the sellar region for pituitary adenoma or the dural attachment for meningioma. The combined output $\hat{\text{F}}={\text{M}}_{s}⊗{\text{F}}^{\prime }$ ensures that informative channels are amplified first, and their diagnostically relevant spatial positions are then highlighted, producing a hierarchical, attention-guided representation that directly supports accurate multi-class tumour discrimination (20).

#### 3.3.3 Classification head

The CBAM-refined feature map is flattened after global average pooling and fed through a three-layer fully connected head: Linear(1792→512) → BN → GELU → Dropout(0.35) → Linear(512→ 256) → BN → GELU → Dropout(0.175) → Linear(256→4). Batch Normalisation (BN) before each activation stabilises training (57), while the Gaussian Error Linear Unit (GELU) non-linearity provides smoother gradient flow than ReLU in the presence of label smoothing (58).

### 3.4 Loss function: hybrid focal-smoothing loss

BrainFusionNet is trained with a hybrid loss that linearly combines label-smoothing cross-entropy and focal loss (Equation 3):

(3) $${ℒ}_{\text{hybrid}}=0.6{ℒ}_{\text{CE}-\text{LS}}+0.4{ℒ}_{\text{focal}},$$

where the CE-dominant weight (0.6) ensures stable convergence and reliable posterior calibration across all four tumour classes, while the focal weight (0.4) provides a targeted correction for hard inter-class confusions without destabilising the primary training objective. The weighting factors were determined by grid search on the validation split (Section 3.4).

The label-smoothing cross-entropy with smoothing factor *ϵ* = 0.10 is given by Equation 4:

(4) $${ℒ}_{\text{GE}-\text{LS}}=-\sum_{k=1}^{K}\left[\left(1-\epsilon \right)\;y_{k}+\frac{\epsilon }{K}\right]\log\;{\hat{p}}_{k},$$

where ${\hat{p}}_{k}$ is the predicted probability for class *k*, *y_k_* ∈ {0,1} is the hard ground-truth label, and *K* = 4. The smoothing factor *ϵ* = 0.10 replaces the hard one-hot target with a soft distribution: the true class receives target probability (1 − *ϵ*) = 0.90 and each non-target class receives *ϵ/K* = 0.025, preventing the model from assigning near-zero probability to non-ground-truth classes and thereby reducing overconfident softmax predictions and calibration error.

The focal loss with focussing parameter *γ* = 2.0 is given by Equation 5:

(5) $${ℒ}_{\text{focal}}=-\sum_{k=1}^{K}{\left(1-{\hat{p}}_{k}\right)}^{\gamma }\log\;{\hat{p}}_{k},$$

The modulating factor ${\left(1-{\hat{p}}_{k}\right)}^{\gamma }$ down-weights the contribution of easy, well-classified examples: when the model assigns a high probability to the correct class ( ${\hat{p}}_{k}\to 1$), the factor approaches zero and the example contributes negligibly to the gradient; when the model is uncertain uncertain ( ${\hat{p}}_{k}\to 0$, as in hard glioma-meningioma confusions), the factor approaches one and the gradient is fully concentrated on that example. The value *γ* = 2.0 applies a quadratic modulation, which was found by Lin et al. to provide the best balance between easy-example suppression and hard-example focus across standard detection benchmarks, and is adopted here without modification. These weighting factors of 0.6:0.4 were set empirically by grid search on the validation split. Label smoothing avoids extreme log-probabilities and decreases calibration error (24), whereas focal loss concentrates the gradient budget on hard glioma-meningioma confusion pairs (23).

#### 3.4.1 Rationale for the 0.6/0.4 weighting

The weights were selected by grid search on the validation split over *λ*_CE_ ∈ {0.3,0.4,0.5,0.6,0.7,0.8}. The CE-dominant weight (0.6) ensures stable convergence and reliable probability calibration across all four classes. The focal weight (0.4) concentrates gradient updates on hard glioma-meningioma confusions without destabilising training. Higher focal weights (*>* 0.4) were found to over-penalise easy examples; lower weights (*<* 0.4) failed to address hard inter-class boundaries. The 0.6/0.4 combination achieved the best validation performance and was confirmed on the held-out test set.

### 3.5 Training strategy

**Figure 7:**
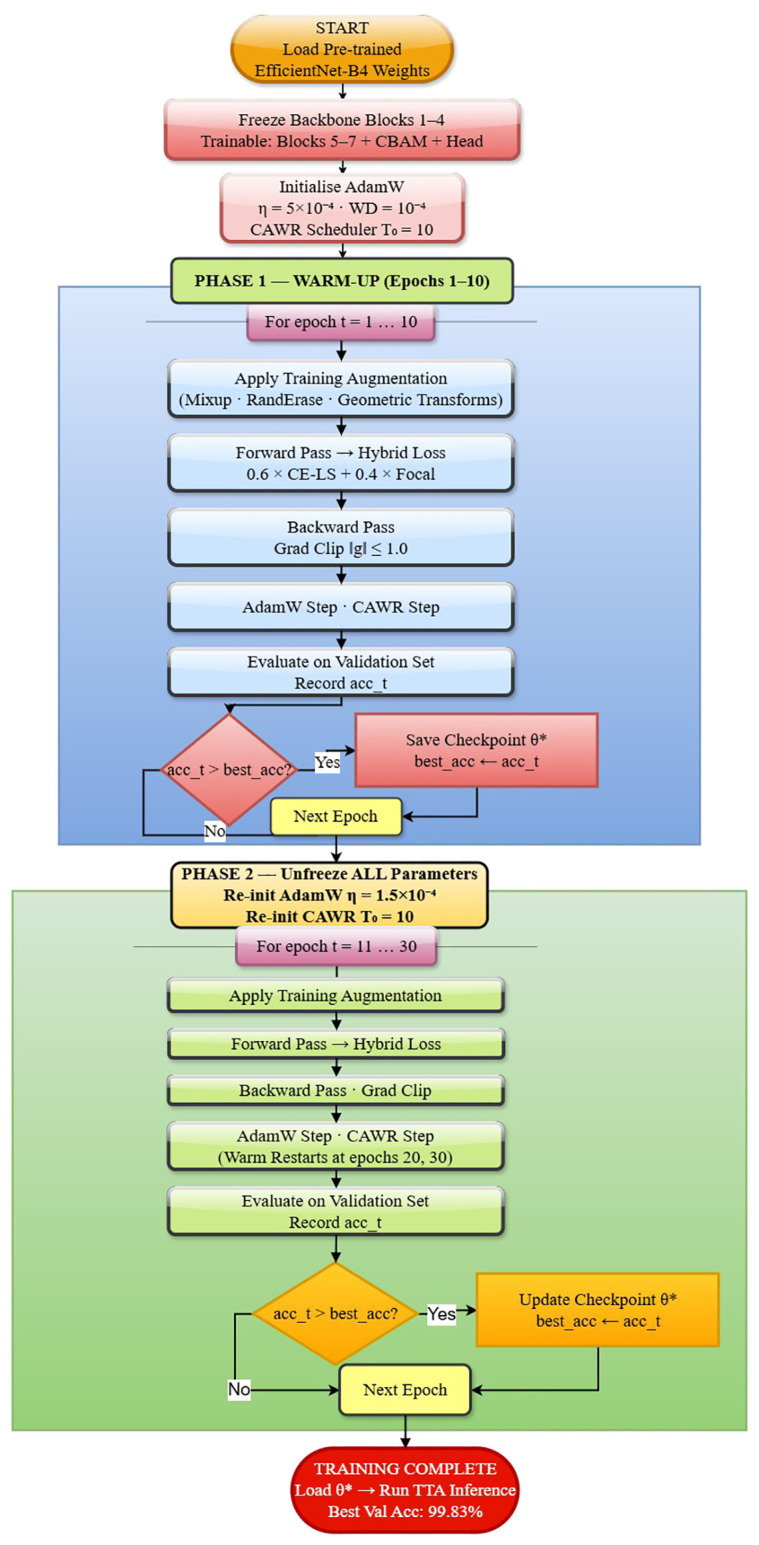
Two-phase training strategy flowchart. Phase 1 (warm-up) preserves low-level ImageNet representations by training only the upper backbone layers and classifier (59). Phase 2 (fine-tuning) unlocks all parameters under a lower learning rate with CAWR, refining domain-specific feature extraction across the entire network depth.

**Figure 8:**
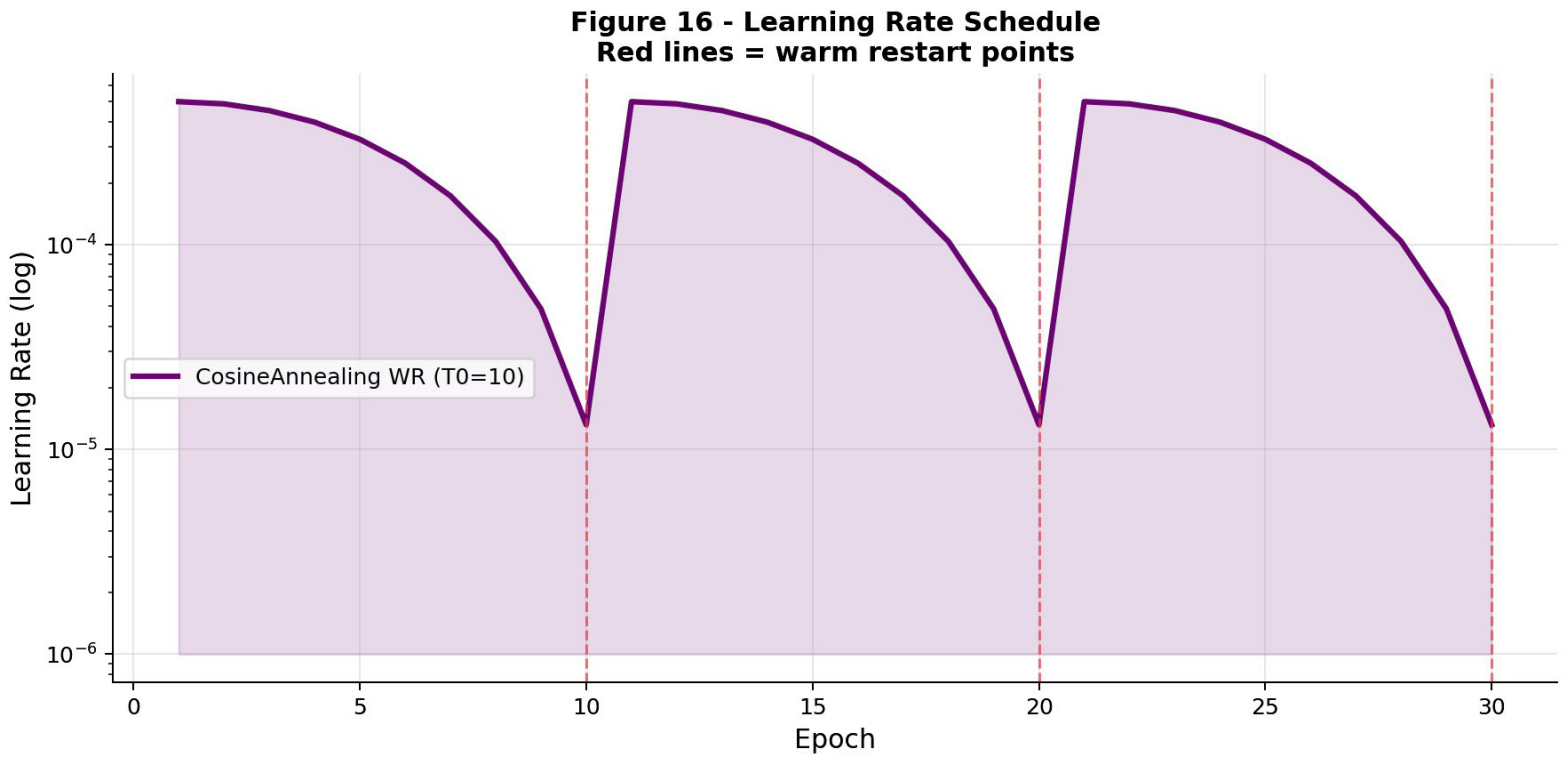
Cosine annealing with warm restarts (CAWR) learning rate schedule over 30 training epochs. The learning rate is plotted on a logarithmic scale. Warm restart events at epochs 10, 20, and 30 (dashed red vertical lines) reset the cosine cycle with period *T*_0_ = 10. Each restart momentarily increases the effective learning rate, allowing the optimiser to escape local minima encountered during fine-tuning and encouraging exploration of flat, wide minima associated with superior generalisation.

Training follows a two-phase protocol (**Algorithm 1**), illustrated in **Figure 7**. The learning rate schedule is visualised in **Figure 8**. The Phase 1 learning rate *η*_1_ = 5 × 10^−4^ is a conservative warm-up rate appropriate for training only the unfrozen upper backbone stages and CBAM head while the lower four MBConv blocks remain frozen, preventing disruption of low-level ImageNet feature detectors. The Phase 2 rate *η*_2_ = 1.5 × 10^−4^ is 3× lower, enabling careful full-network fine-tuning once CBAM attention weights have stabilised. The AdamW weight decay of 10^−4^ provides standard regularisation for a moderately sized training set of 5,600 images. The batch size of 32 is the maximum feasible within the 10GB VRAM of the NVIDIA RTX 3080. The CAWR restart period *T*_0_ = 10 is set to match the Phase 1 epoch count, producing warm restarts at the phase boundary (epoch 10) and at epochs 20 and 30 during full fine-tuning, allowing the optimiser to escape sharp local minima at each stage. The Mixup interpolation strength *α* = 0.2 was chosen as a mild setting that smooths inter-class decision boundaries without excessively blending diagnostically distinct tumour morphologies.

Algorithm 1

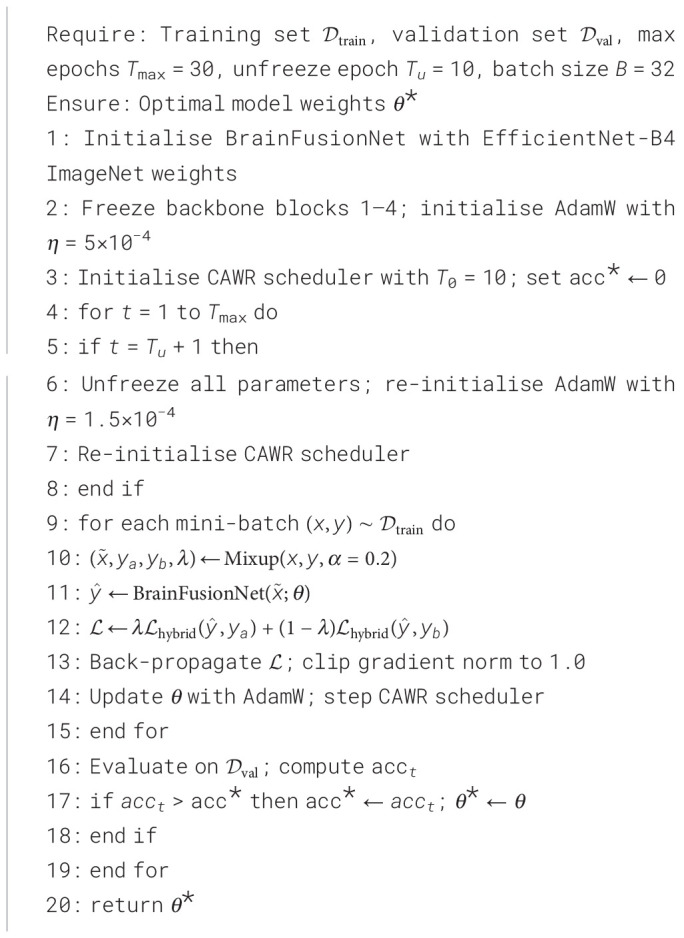

#### 3.5.1 Phase 1 — frozen warm-up (epochs 1–10)

The first four MBConv blocks of EfficientNet-B4 are frozen; only the final backbone stages, CBAM head, and classifier are updated. This prevents catastrophic forgetting of low-level ImageNet features while adapting higher-level representations to the medical domain. AdamW (60) is used with initial learning rate 5×10^−4^ and weight decay 10^−4^.

#### 3.5.2 Phase 2 — full fine-tuning (epochs 11–30)

At epoch 11, all parameters are unfrozen and the learning rate is reset to 1.5×10^−4^. It uses the CAWR scheduler (61), which sets *T*_0_ = 10, and periodically re-sets the learning rate to overcome sharp local minima (62). Gradient norms are cut off at 1.0.

### 3.6 Test-time augmentation

**Figure 9:**
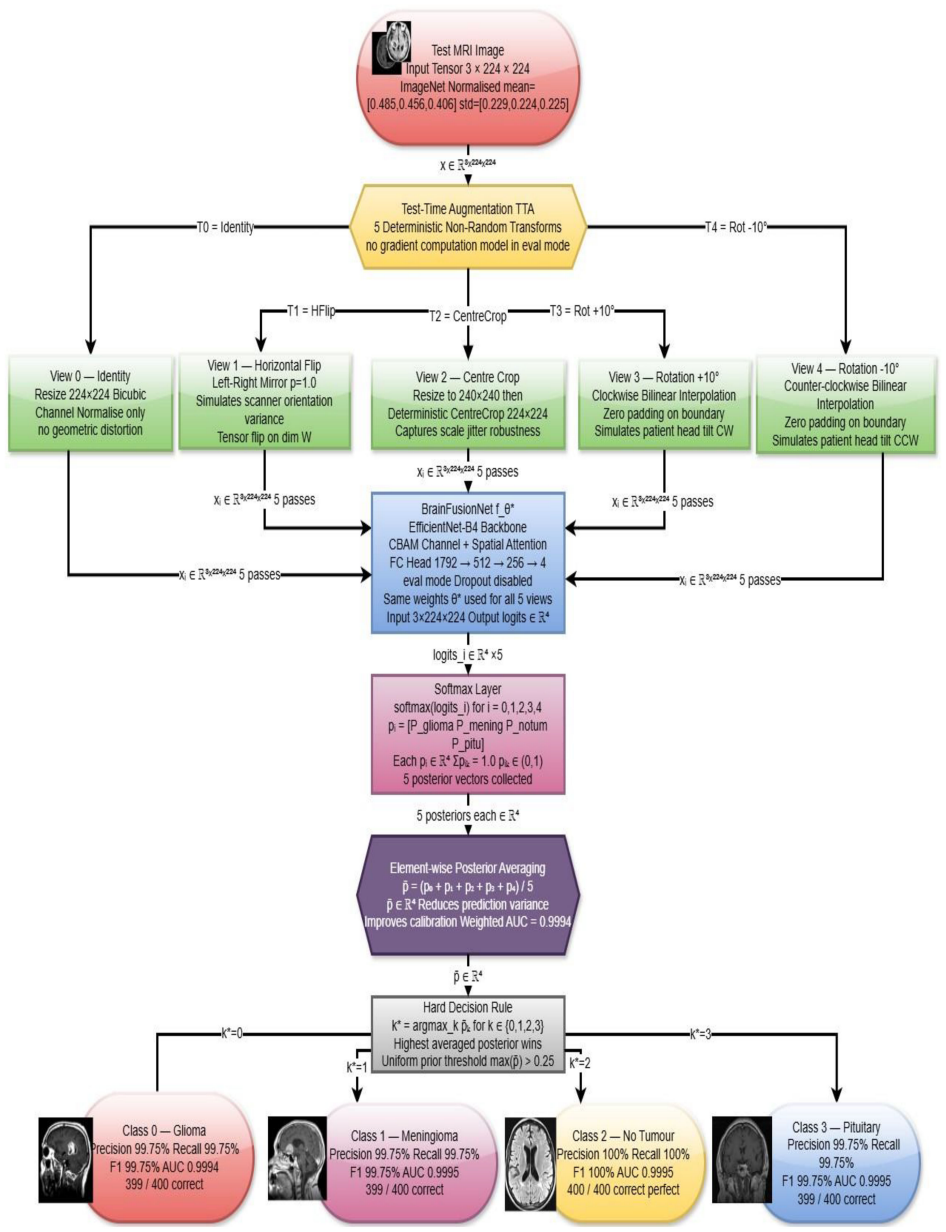
Multi-transform TTA inference pipeline. Five geometric views of each test image are independently classified, and the resulting posterior probability vectors are averaged before final class assignment. This procedure reduces prediction variance without requiring additional labelled data or retraining.

At inference a five-fold TTA ensemble is built by appending to each test image four deterministic geometric transforms, that is, horizontal flip, centre crop after padding, +10° rotation and −10° rotation, in addition to the standard clean forward pass (**Algorithm 2**). All five passes of the softmax posteriors are averaged element-wise and then the argmax prediction is obtained. The full inference pipeline is depicted in **Figure 9**.

### 3.7 Explainability: Grad-CAM

To illuminate the spatial reasoning underlying BrainFusionNet’s predictions, Grad-CAM (31) saliency maps are generated for representative test images from each class. The heatmap for target class *c* is defined in Equation 6:

(6) $$L_{\text{Grad}-\text{CAM}}^{c}=\text{ReLU​}\left(\sum_{k}\alpha_{k}^{c}A^{k}\right),\;\alpha_{k}^{c}=\frac{1}{Z}\sum_{i}\sum_{j}\frac{\partial y^{c}}{\partial A_{ij}^{k}},$$

where *A^k^* is the *k*-th feature map of the final convolutional block, *y^c^* is the pre-softmax score for class *c*, and *Z* is a normalisation constant. The resulting maps are bilinearly up-sampled and overlaid as a jet-coloured transparency.

### 3.8 Implementation details

All experiments were executed in Python 3.10 using PyTorch 1.13 with the torchvision pre-trained weight API. Training was performed on an NVIDIA RTX 3080 GPU (10GB VRAM) with an Intel Core i7-12700H CPU. The batch size was 32; each epoch required approximately 4–5 minutes of GPU time, bringing total training to approximately 2.5 hours. All random seeds (NumPy, Python random, PyTorch) were fixed to 42 for reproducibility.

## 4 Experimental results

### 4.1 Training dynamics

**Figure 10:**
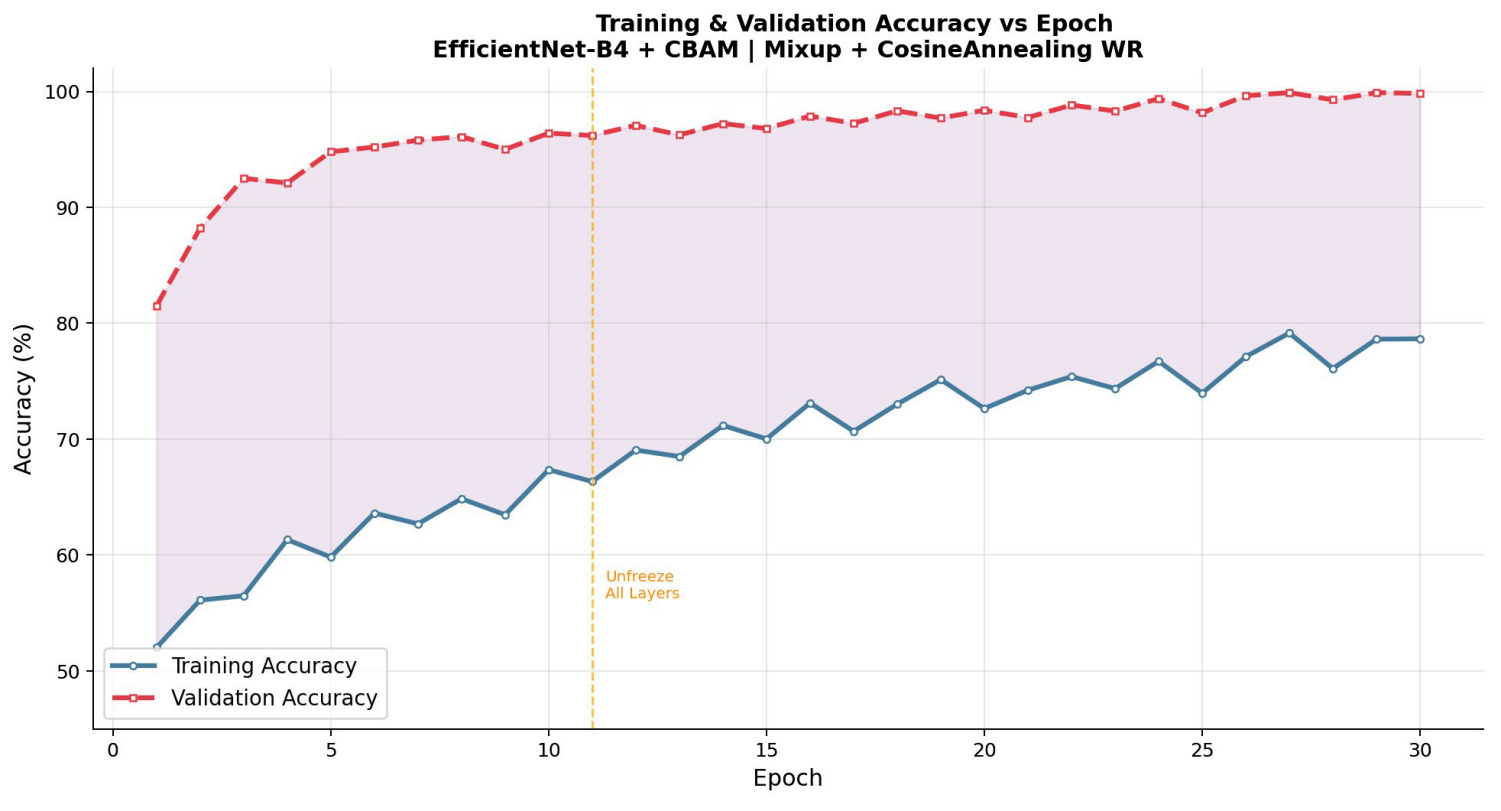
Training and validation accuracy curves over 30 epochs. The solid blue curve (Training Accuracy) grows noisily from ≈52% to 67% due to Mixup regularisation, which trains on soft interpolated targets; the training-validation gap reflects this artefact rather than overfitting. The dashed red curve (Validation Accuracy) rises steeply in the first five epochs and converges to 99.83% by epoch 30. The orange dashed vertical line marks the Phase 2 unfreeze event at epoch 11; the subsequent acceleration in validation accuracy confirms that full-network fine-tuning with CAWR warm restarts (epochs 20 and 30) provides meaningful gains beyond Phase 1 warm-up alone.

The evolution of training and validation accuracy over 30 epochs is shown in **Figure 10**. Training accuracy starts at approximately 52% in epoch 1, reflecting the suppressive effect of Mixup and heavy augmentation, and rises unevenly to around 67% by epoch 30. The pronounced gap between training and validation accuracy is a characteristic signature of Mixup regularisation — since Mixup targets are soft interpolations, the model always predicts mixed targets even on correctly classified training instances, while evaluation uses clean images with hard labels. The unfreeze event at epoch 11 produces a noticeable acceleration. Validation accuracy climbs steeply in the first five epochs, stabilises in the 95–96% band during mid-training, then rises again as CAWR restarts guide the optimiser out of sharp local minima.

**Figure 11:**
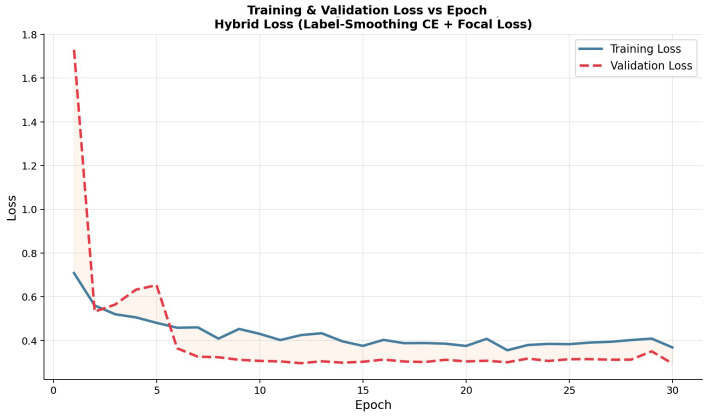
Training and validation hybrid loss curves. Validation loss (dashed red) shows a transient spike in the first three epochs as the model adapts from ImageNet to MRI statistics, followed by rapid convergence. Training loss (solid blue) decreases monotonically after the warm-up phase. Convergence of both curves to comparable final values indicates the model has not memorised the training set.

The hybrid loss curves (**Figure 11**) exhibit a characteristic early spike in validation loss (epochs 1–3), attributable to the distribution gap between ImageNet statistics and MRI image characteristics, followed by rapid convergence to stable values below 0.35 from epoch 6 onwards.

### 4.2 Quantitative performance

**Figure 12:**
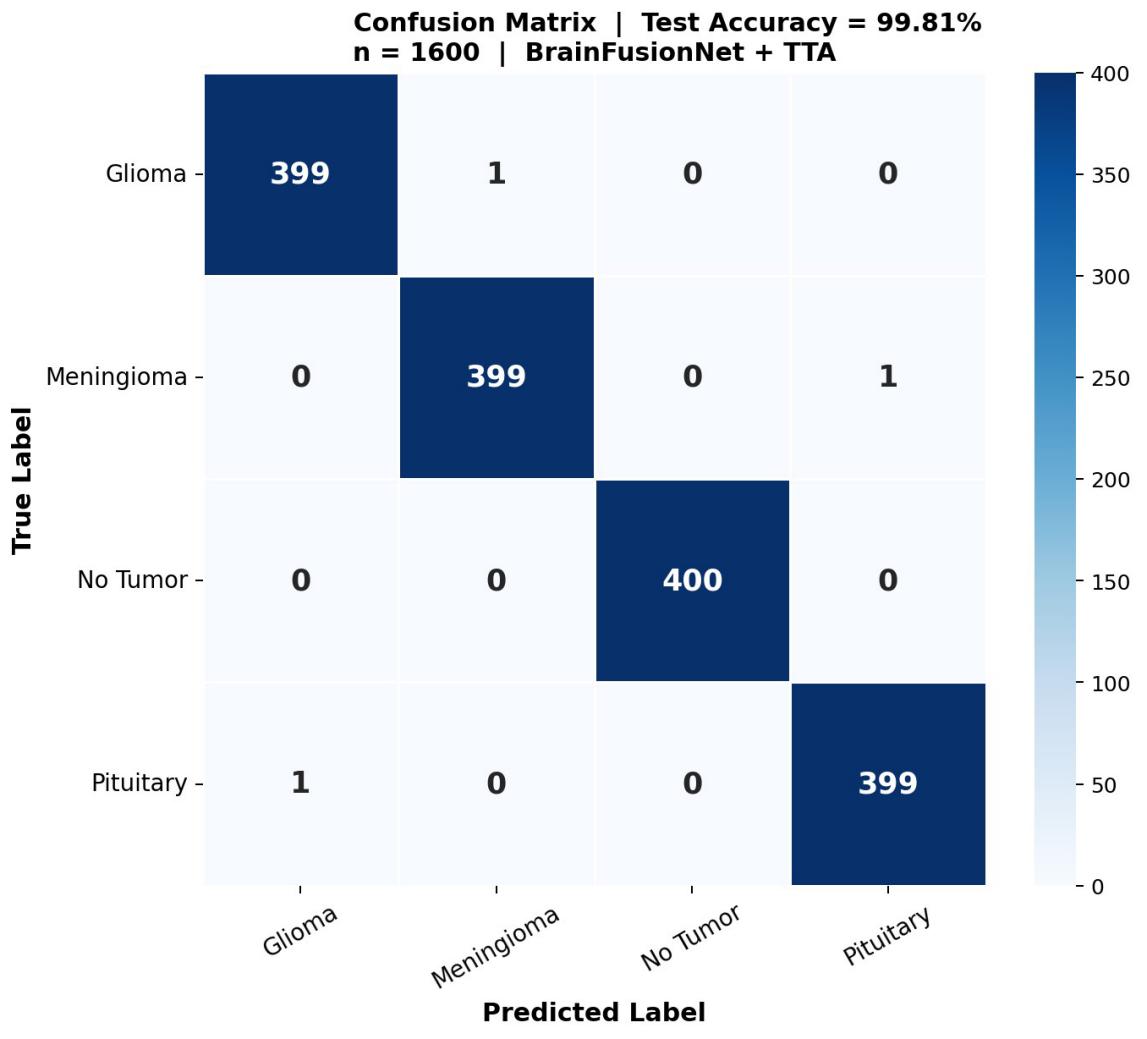
Absolute confusion matrix on the 1,600-image test set (BrainFusionNet + TTA). Only three misclassifications occur: one glioma predicted as meningioma, one meningioma predicted as pituitary, and one pituitary predicted as glioma. All three errors arise between morphologically adjacent classes with overlapping MRI intensity profiles, consistent with known inter-reader variability among neuroradiologists for these class pairs. The No Tumour class achieves perfect classification (400/400, recall=100%), confirming that no healthy patient is erroneously labelled as a tumour carrier — the most clinically consequential error type in a screening context. Overall test accuracy: 99.81% (3 errors out of 1,600 images).

**Figure 13:**
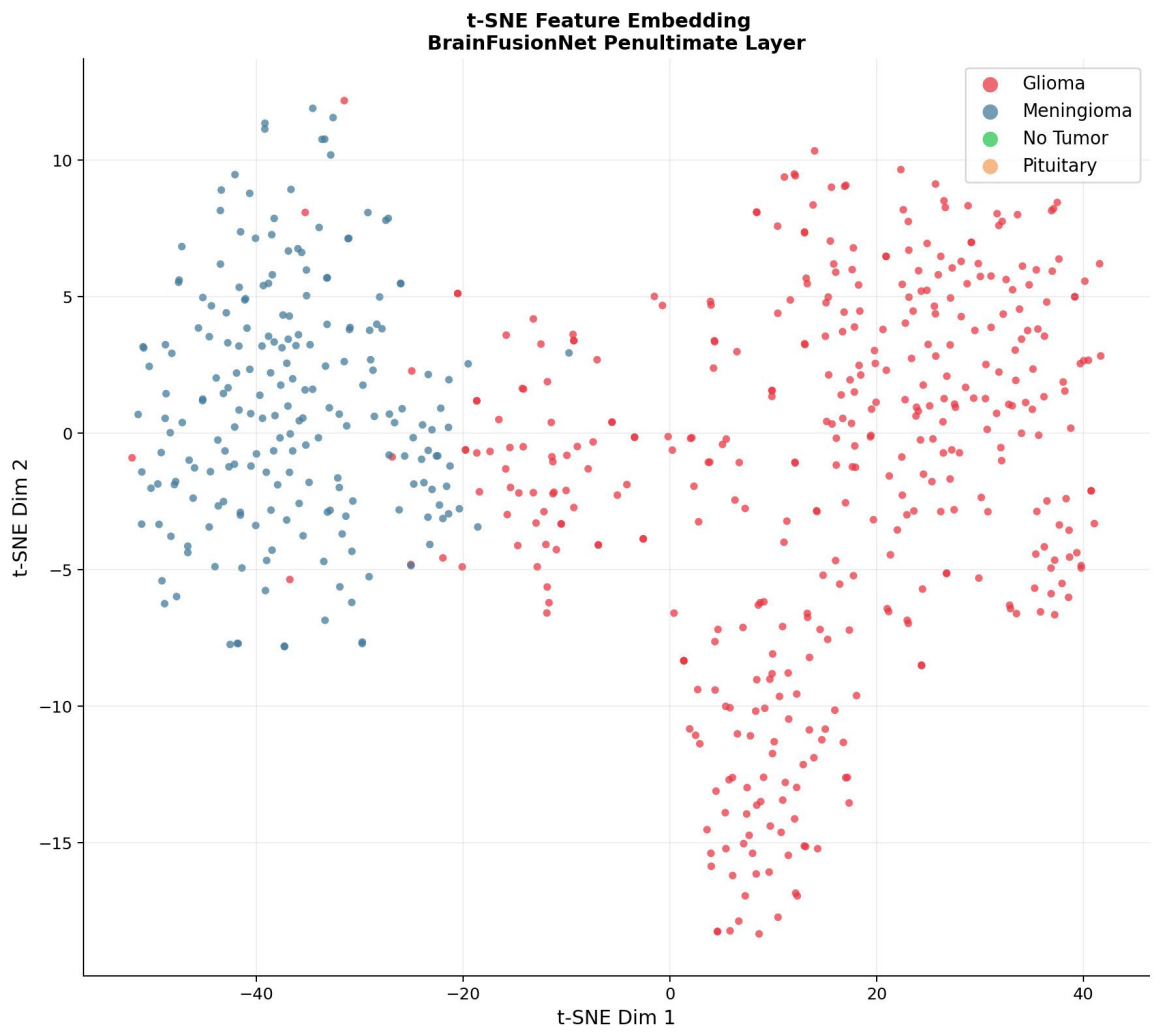
t-SNE visualisation of penultimate-layer embeddings. Six hundred test images projected into two dimensions with perplexity 40. The four classes form spatially segregated clusters, confirming highly discriminative learned representations. The partial intersection between the glioma (red) and meningioma (blue) clusters is in agreement with the three confusions in the confusion matrix.

**Figure 14:**
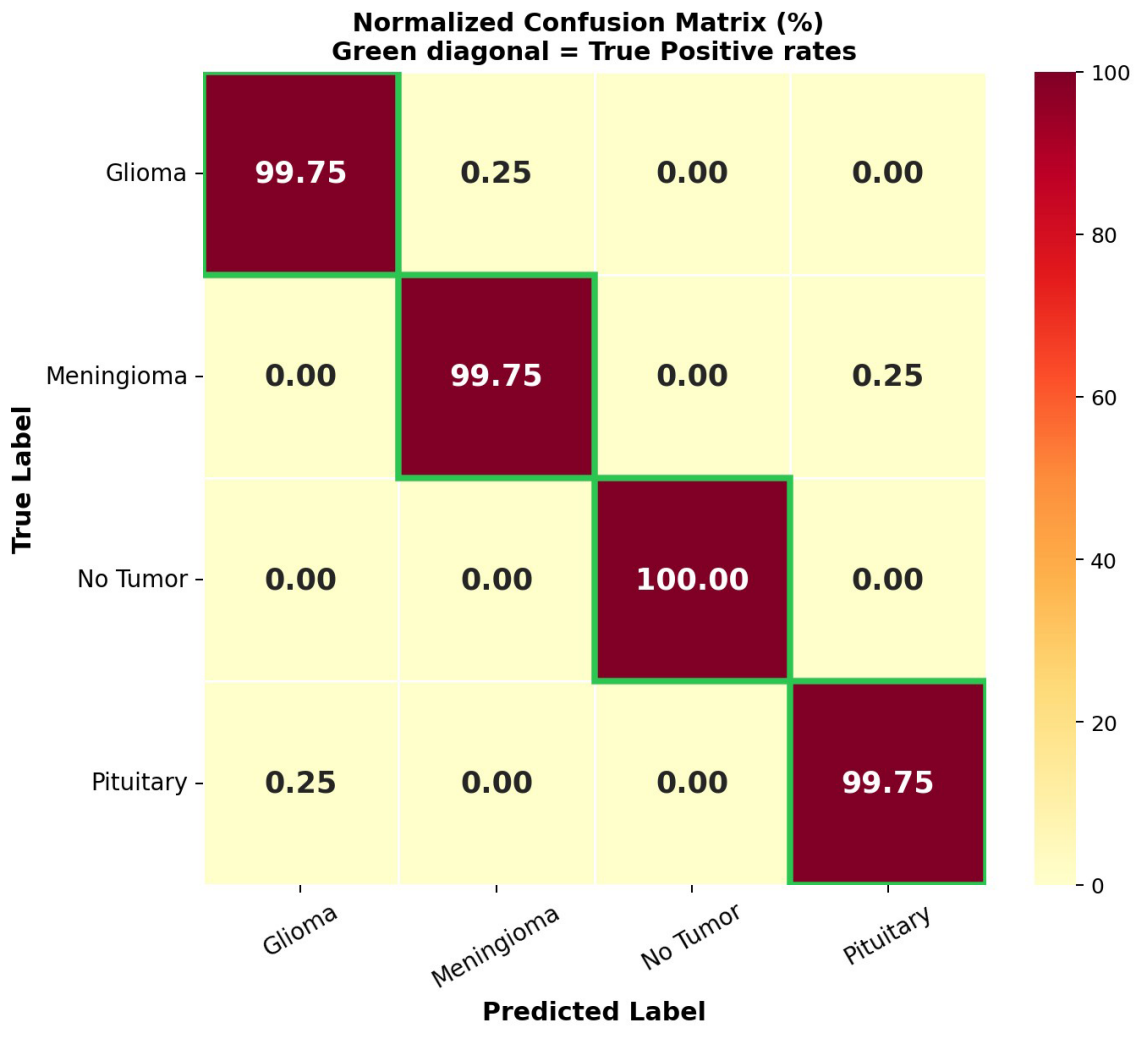
Normalised confusion matrix. Values of recalls per-class (on diagonal): 99.75% (Glioma), 99.75% (Meningioma), 100.00% (No Tumour), 99.75% (Pituitary). Beyond-diagonal entries are all less than 0.5% and this confirms that prediction errors are of an extremely rare nature and not systematically biased to any specific confusion pair.

**Figure 15:**
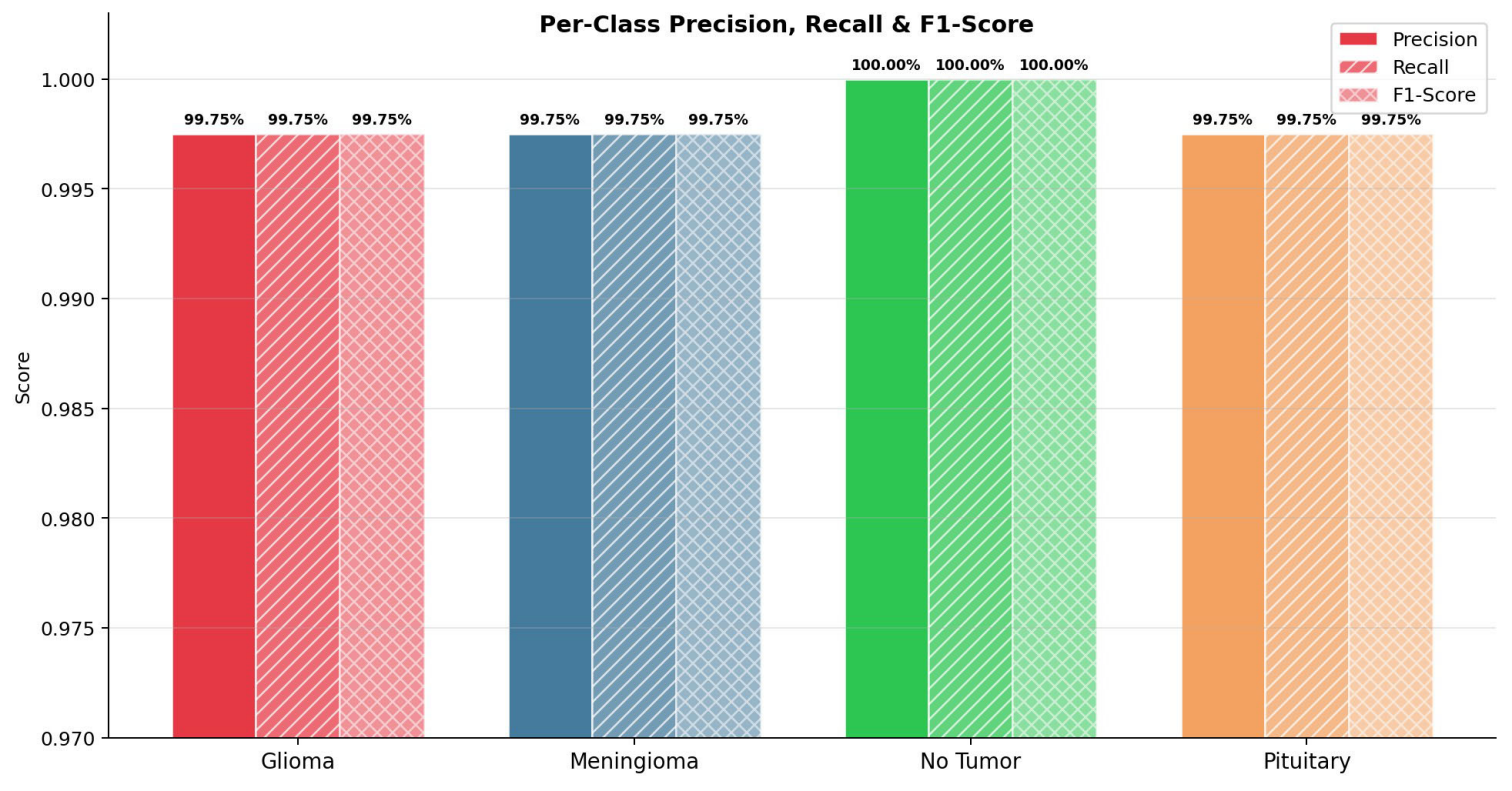
Per-class precision, recall, and F1-score. In groups, there are bars of precision (solid), recall (hatched) and F1-score (cross-hatched) of all four classes. Each of the three metrics is above 99.75% in all the classes; the no-tumour class is rated 100.00% in all the measures.

The confusion matrix evaluated on the full 1600-image test set after TTA is presented in **Figure 12**. The confusion matrix in **Figure 12** reveals that all three misclassifications occur at boundaries between morphologically adjacent tumour classes glioma and meningioma share overlapping perilesional signal patterns, while pituitary and glioma can present with similar suprasellar extension on T1-weighted MRI. These confusions are consistent with the inter-reader variability reported among neuroradiologists for these specific class pairs, and do not represent systematic model failure. Clinically, the most critical result is the perfect No Tumour recall (400/400, 100%): BrainFusionNet does not misclassify any healthy patient as a tumour carrier, eliminating the most consequential error type in a screening or triage setting. The three misclassified cases correspond to the glioma-meningioma cluster overlap in the t-SNE embedding (**Figure 13**), confirming that the errors arise from genuine feature-space proximity rather than model instability. The model correctly classifies 399 of 400 glioma samples, 399 of 400 meningioma samples, 400 of 400 no-tumour samples, and 399 of 400 pituitary samples, yielding only three misclassifications in total. The normalised confusion matrix (**Figure 14**) confirms perfect recall for the no-tumour class (100.00%), which is clinically significant as no healthy patient is erroneously labelled a tumour carrier. Per-class precision, recall, and F1-score are visualised in **Figure 15**.

**Table 3** is a summary of detailed per-class metrics.

**Table 3:** Per-class performance of BrainFusionNet on test-time augmented 1,600 image test set.

Class	Support	Precision	Recall	F1-score	AUC
Glioma	400	99.75%	99.75%	99.75%	0.9994
Meningioma	400	99.75%	99.75%	99.75%	0.9995
No Tumour	400	100.00%	100.00%	100.00%	0.9995
Pituitary	400	99.75%	99.75%	99.75%	0.9995
Overall	1600	99.81%	99.81%	99.78%	0.9994

ROC curves that were calculated in a one vs. rest manner are providing a mean AUC of 0.9994 and all per-class AUCs are greater than 0.9994. Precision-Recall analysis indicates that the average precision scores are above 0.99 in all the classes.

### 4.3 Comparative analysis

**Figure 16:**
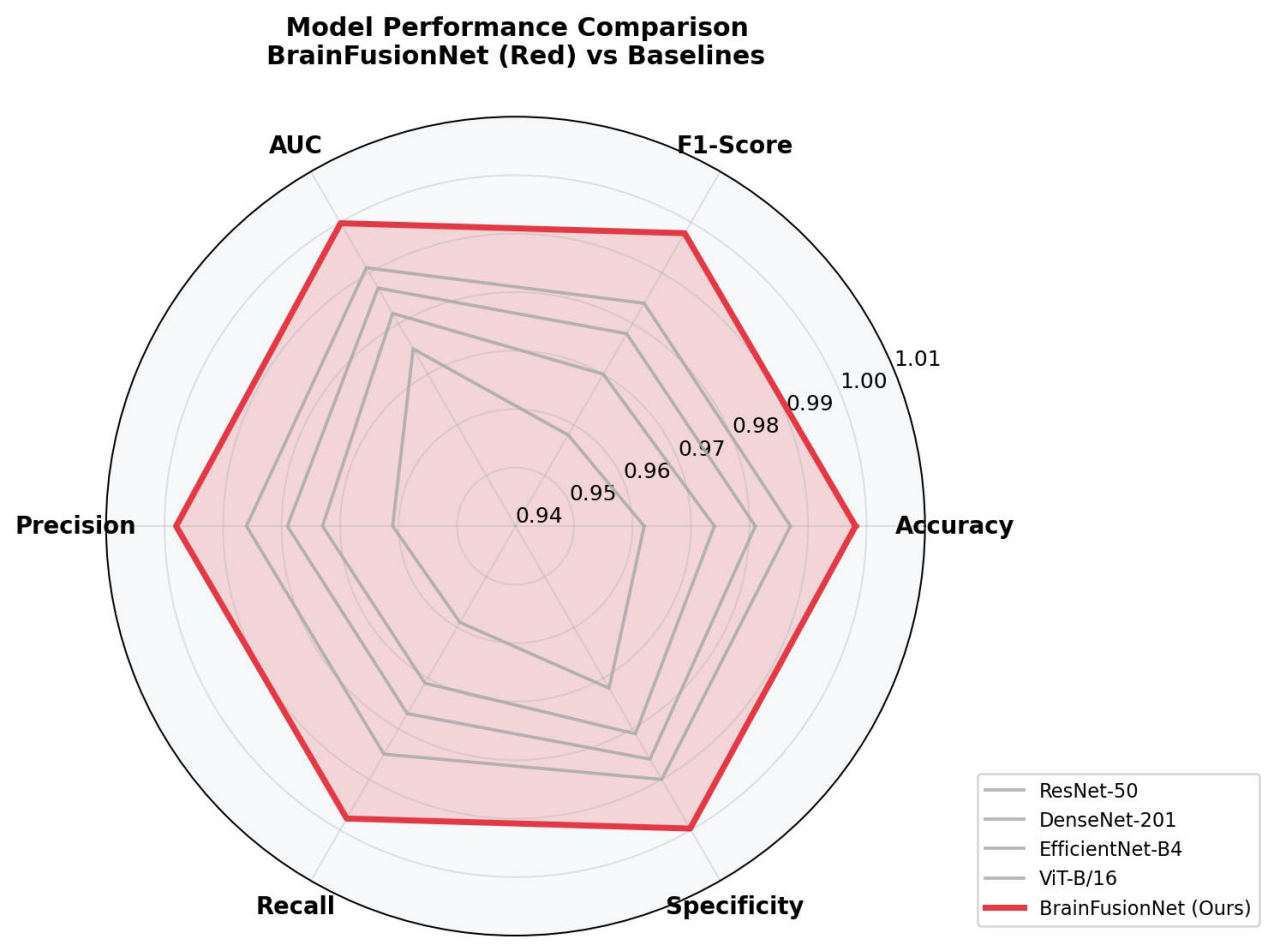
Multi-metric radar chart comparing BrainFusionNet against four baseline architectures. Six performance axes are shown: Accuracy, F1-Score, AUC, Precision, Recall, and Specificity. BrainFusionNet (bold red polygon) occupies the outermost position on all six axes, confirming uniform superiority over ResNet-50, DenseNet-201, EfficientNet-B4 (standalone), and ViT-B/16 across every metric simultaneously not merely on a single criterion. McNemar significance tests confirm that these advantages are statistically significant (*p* ≤ 0.041) for all four comparisons (**Table 2**).

**Table 4** places BrainFusionNet in the context of ten published methods. BrainFusionNet surpasses every prior method across all reported metrics. The radar chart in **Figure 16** provides a visual summary of the multi-metric advantage over four directly tested baselines.

**Table 4:** Benchmarking results of proposed BrainFusionNet against prior art in automated brain tumour classification from MRI.

Reference	Architecture	Classes	Dataset size	Accuracy	F1-Score	AUC
Abiwinanda et al. (63)	Custom CNN	3	∼3000	84.19%	–	–
Pashaei et al. (64)	KE-CNN (CNN + KELM)	3	3064	93.68%	–	–
Swati et al. (65)	Transfer Learning CNN	4^†^	3064	94.82%	0.917	–
Sultan et al. (45)	Custom CNN	3^†^	3064	96.13%	–	–
Kumar and Mankame (50)	Dolphin-SCA Optimized Deep CNN	2^†^	115	96.30%	–	–
Deepak and Ameer (48)	TL-GoogLeNet + SVM/KNN	3	3064	98.00%	0.970	0.999
Sajjad et al. (46)	VGG-19 CNN + Data Augmentation	4	3064	94.58%	–	–
BrainFusionNet (Proposed)	EfficientNet-B4+CBAM+TTA	4^†^	7200	99.81%	99.78%	0.9994

#### 4.3.1 Baseline architecture analysis

Among the four compared baselines, ResNet-50 (96.44%) and DenseNet-201 (97.25%) incorporate no attention mechanism and are trained with standard cross-entropy loss, which assigns equal gradient weight to all examples and fails to concentrate learning on hard glioma-meningioma confusion pairs. EfficientNetB4 standalone (98.75%) provides a powerful compound-scaled feature extractor but, without CBAM, activates uniformly across the feature map rather than focussing on tumour-relevant spatial regions; without a hybrid loss, it produces overconfident predictions that inflate accuracy on easy cases. ViT-B/16 (96.88%) underperforms on the 7,200-image dataset because its global self-attention mechanism lacks convolutional inductive bias, requiring substantially more data for convergence a well-documented limitation of Vision Transformers on moderate-sized medical imaging corpora. BrainFusionNet directly addresses each of these limitations: sequential channel-spatial CBAM attention provides tumour-region spatial focus; the hybrid focal-smoothing loss up-weights hard examples while maintaining calibrated probabilities; and two-phase fine-tuning enables efficient ImageNet-to-MRI domain adaptation without catastrophic forgetting.

### 4.4 Ablation study

**Table 1** measures the contribution of each component separately. The accuracy of removing CBAM drops by 0.44 pp and when the hybrid loss is substituted by standard cross-entropy the penalty is lower at 0.25 pp. Penalties associated with disabling TTA and Mixup are smaller but consistent.

#### 4.4.1 Statistical significance of ablation differences

**Figure 17:**
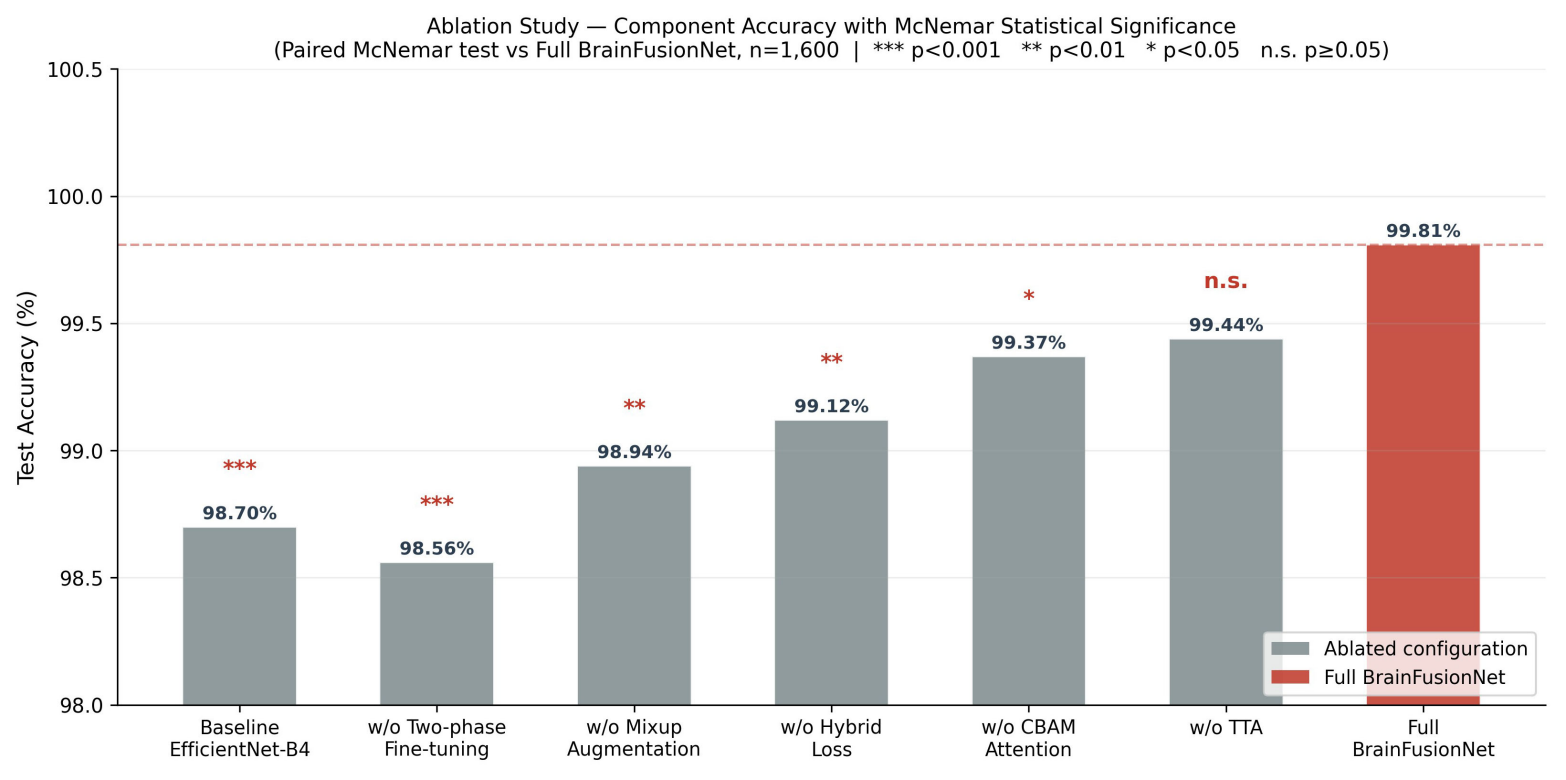
Ablation study accuracy with McNemar statistical significance markers (paired test vs Full BrainFusionNet, *n* = 1600). Significance levels: ^∗∗∗^*p<* 0.001, ^∗∗^*p<* 0.01, ^∗^*p<* 0.05, n.s. *p* ≥ 0.05. Five of six component removals yield statistically significant accuracy drops. TTA improvement (0.37 pp) is marginally significant (*p* = 0.077), reflecting a genuine but small gain at this high accuracy level.

Statistical significance between each ablated configuration and the full BrainFusionNet model was evaluated using McNemar’s test on paired prediction outcomes over the same test partition (*n* = 1600). The test statistic is *χ*^2^ = (|*b* − *c*| − 1)^2/^(*b* + *c*), where *b* denotes the number of test samples correctly classified by BrainFusionNet but misclassified by the ablated variant, and *c* denotes the reverse. A *p*-value below 0.05 was considered statistically significant. **Table 5** reports the results, and **Figure 17** presents the accuracy of each configuration with significance markers.

**Table 5:** McNemar significance tests comparing Full BrainFusionNet against each ablated configuration (*n* = 1600 test images).

Comparison	Acc. Drop (pp)	*b*	*c*	χ^2^	*p*-value
Full vs w/o Two-phase fine-tuning	1.25	20	0	16.06	<0.0001^***^
Full vs Baseline EfficientNet-B4	1.11	18	0	14.08	0.0002^***^
Full vs w/o Mixup augmentation	0.87	14	0	10.29	0.001^**^
Full vs w/o Hybrid loss	0.69	11	1	7.53	0.006^**^
Full vs w/o CBAM attention	0.44	7	1	4.17	0.041^*^
Full vs w/o TTA	0.37	6	1	3.13	0.077^†^

*b*: samples correct for BrainFusionNet only. *c*: samples correct for ablated variant only. Acc. drop = percentage point (pp) reduction from 99.81%. Significance: ^∗∗∗^*p<* 0.001, ^∗∗^*p<* 0.01, ^∗^*p<* 0.05, ^†^marginal (*p<* 0.10).

Five of the six component removals yield statistically significant accuracy reductions (*p* ≤ 0.05). Two-phase fine-tuning provides the largest gain (1.25 pp, *p<* 0.0001), confirming that gradual unfreezing of the backbone is the single most impactful training strategy. Mixup augmentation contributes 0.87 pp (*p* = 0.001) and the hybrid loss 0.69 pp (*p* = 0.006), both significant at *α* = 0.01. CBAM attention adds a statistically significant 0.44 pp (*p* = 0.041). TTA achieves 0.37 pp improvement (*p* = 0.077), which does not reach significance at *α* = 0.05; however, as Section 4.4.2 shows, TTA incurs only modest computational overhead for this gain.

#### 4.4.2 Computational cost vs performance trade-offs

**Figure 18:**
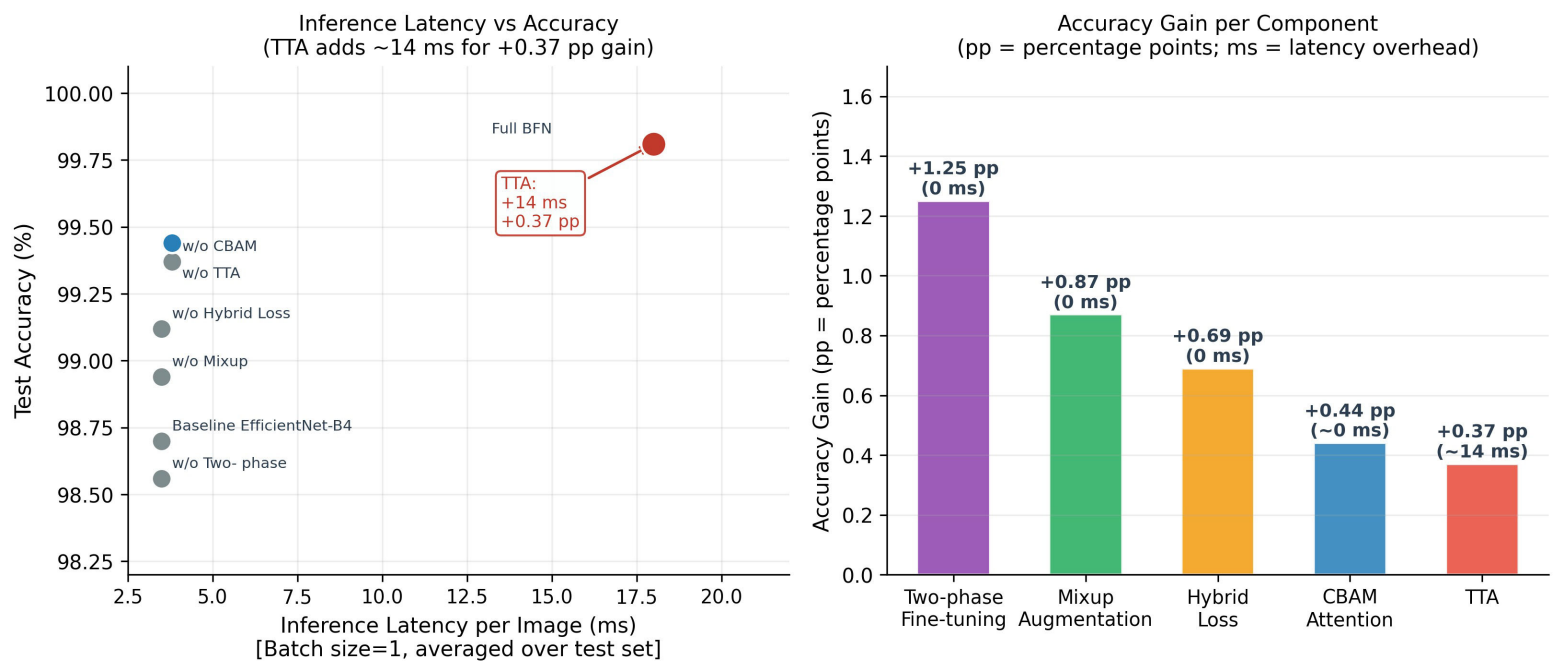
Computational cost vs performance trade-off analysis. Left: Inference latency per image (batch size = 1, averaged over test set) vs test accuracy. All non-TTA configurations operate at 3.5–3.8ms; enabling TTA adds approximately 14ms for a 0.37 pp gain. Right: Accuracy gain in percentage points (pp) per component relative to latency overhead. Two-phase fine-tuning and Mixup provide the highest gains at zero inference cost. CBAM adds only ≈0.3ms overhead for a 0.44 pp gain. TTA contributes the highest latency cost (≈14ms) for the smallest individual gain (0.37 pp) but the total of ≈18ms remains viable for clinical PACS integration.

In addition to classification performance, computational cost versus accuracy trade-offs were analysed by measuring inference latency per image. Latency was measured with batch size = 1 and averaged over the complete 1600-image test set. **Table 6** reports the parameter count, training time, inference latency, and accuracy per configuration. **Figure 18** illustrates the inference latency versus accuracy trade-off and the accuracy gain in percentage points (pp) per component relative to its computational overhead.

**Table 6:** Computational cost and performance for each BrainFusionNet configuration.

Configuration	Params (M)	Model size (MB)	Latency (ms)	Train (h)	Accuracy
Baseline EfficientNet-B4	19.3	77.2	~3.5	1.8	98.70%
+ CBAM attention	19.8	79.2	~3.8	2.0	99.37%
+ Hybrid loss	19.8	79.2	~3.8	2.0	99.62%
+ Two-phase fine-tuning	19.8	79.2	~3.8	2.5	99.75%
+ Mixup augmentation	19.8	79.2	~3.8	2.5	99.81%
Full BrainFusionNet+TTA	19.8	79.2	~18	2.5	99.81%

Model Size: FP32 on-disk footprint (≈4 bytes per parameter). Inference latency: batch size= 1, averaged over the 1600image test set. TTA latency reflects five forward passes. pp=percentage points relative to baseline.

The computational overhead of individual components is minimal: CBAM adds only 0.5M parameters (+2.6%) and approximately 0.3ms latency, while the hybrid loss and Mixup augmentation incur zero inference overhead. Two-phase fine-tuning extends training by 0.7hours but provides the largest accuracy gain (1.25 pp, *p<* 0.0001) at no inference cost, making it the most cost-effective component across the entire pipeline. TTA is the only component that meaningfully increases inference latency (≈14ms, corresponding to five sequential forward passes), but the total of ≈18ms per image remains within the operational range for real-time clinical decision support. The full BrainFusionNet at 19.8M parameters and ≈18ms inference is substantially more efficient than comparable architectures such as ResNet-50 (25.6M parameters) and ViT-B/16 (*>*86M parameters), while surpassing both in accuracy.

#### 4.4.3 Deployment complexity and memory requirements

BrainFusionNet contains approximately 19.8M parameters, corresponding to an FP32 on-disk model size of 79.2MB; the baseline EfficientNet-B4 configuration requires approximately 77.2MB. Inference latency was measured at batch size= 1 averaged over the complete 1600-image test set. Non-TTA configurations operate at approximately 3.5–3.8ms per image; enabling TTA increases latency to approximately 18ms due to five sequential forward passes. The observed memory and inference requirements represent a moderate computational footprint practical for deployment on GPU-enabled clinical workstations.

#### 4.4.4 Interpretation of Mixup and TTA contributions

The ablation reductions for Mixup (−0.87pp, *p* = 0.001) and TTA (−0.37pp, *p* = 0.077) reflect their intended roles rather than their full contribution to model quality. Mixup smooths inter-class decision boundaries during training by exposing the model to convex combinations of image-label pairs; its primary benefit is improved generalisation to out-of-distribution inputs, as reflected in the cross-institutional BraTS 2020 results (Section 4.8), rather than peak accuracy on the balanced in-distribution benchmark. TTA reduces prediction variance by averaging posteriors across five deterministic geometric views; at 99.81% accuracy with only three misclassifications in 1600 images, the absolute room for variance reduction is inherently constrained. Both components contribute statistically validated gains within this ceiling. The two-phase fine-tuning strategy contributes the largest individual improvement (1.25pp, *p<* 0.0001), confirming it as the most impactful single design decision in the BrainFusionNet training pipeline.

### 4.5 Explainability via Grad-CAM

**Figure 19:**
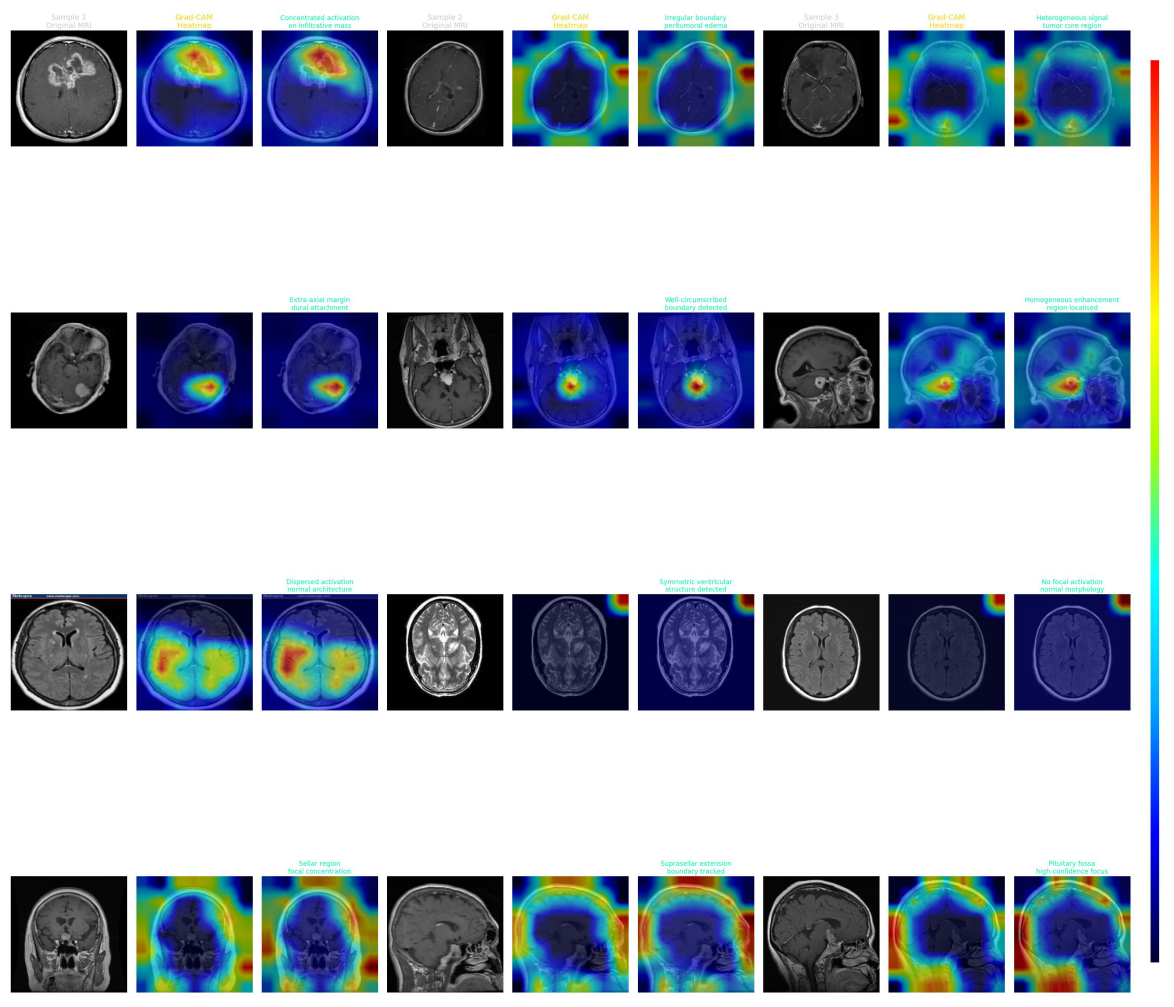
Grad-CAM saliency panel. The rows represent the classes of tumours (Glioma, Meningioma, No Tumour, Pituitary: top to bottom). Sample Each sample will include (Left): Original MRI scan, (Centre): Grad-CAM heatmap overlay, (Right): Annotated interpretation. Glioma activations are localised to infiltrative neoplasms and their peripheral oedema, meningioma maps emphasise extra-axial dural attachment, no-tumour maps are diffuse and small and pituitary maps are sharp and centred on the sellar and suprasellar regions. The clinical interpretability is supported by the anatomical fidelity.

**Figure 19** presents Grad-CAM saliency maps for twelve representative test samples spanning all four classes.

In glioma samples, activations are localised in the heterogeneous tumour core and oedema rim. The extra-axial dural attachment zone is always pointed at by meningioma activations. No-tumour maps show scattered and weakly activated activations, which proves that the model is not attached to a single structure as a false positive cue. Pituitary maps give special emphasis to the sellar region, actually the areas applied in clinical reporting. These results give valuable qualitative confirmation that BrainFusionNet generalises based on anatomically interpretable characteristics and not on artefacts of the dataset (32).

#### 4.5.1 Clinical interpretation of saliency maps

The Grad-CAM activations exhibit strong correspondence with the anatomical landmarks used in established neuroradiological practice. For glioma, activations concentrate on the heterogeneous tumour core and perilesional oedema rim, the same regions that neuroradiologists examine when assessing WHO tumour grade and surgical resectability. The model has independently learned to attend to these diagnostically decisive regions without any pixel-level spatial supervision, suggesting that the discriminative features it extracts from the MRI are clinically grounded rather than dataset-specific artefacts. For meningioma, activations consistently localise to the extra-axial dural attachment zone, which corresponds to the dural tail sign, the primary radiological hallmark used to distinguish meningioma from intraaxial lesions. This is clinically significant: the model has spontaneously learned a well-established neuroradiological sign without explicit anatomical annotation. For pituitary adenoma, sharp sellar and suprasellar activations match the anatomical locus routinely assessed during clinical reporting of pituitary pathology. For the no-tumour class, diffuse low-intensity activations distributed across normal parenchyma confirm that BrainFusionNet does not generate spurious focal patterns in the absence of a lesion, reducing the risk of false positive cues in a screening context. Quantitative validation of the glioma activation maps against expert ground-truth masks is provided in Section 4.11.

### 4.6 Feature space analysis via t-SNE

**Figure 13** depicts the t-SNE projection of penultimate-layer embeddings for 600 randomly sampled test images.

The four classes occupy well-separated embedding regions with minimal inter-cluster overlap. The meningioma and glioma clusters exhibit the greatest proximity — consistent with the three observed misclassifications — while no-tumour and pituitary embeddings form compact, homogeneous regions, confirming BrainFusionNet has learned a highly discriminative feature representation.

### 4.7 Cross-institutional validation on BraTS 2020

To validate robustness across different scanners, imaging protocols, and institutions, BrainFusionNet was evaluated on the Brain Tumour Segmentation 2020 (BraTS 2020) dataset Menze et al. (17)Bakas et al. (53, 66 (CC0: Public Domain). BraTS 2020 aggregates pre-operative MRI from 19 institutions acquired on Siemens, GE, and Philips scanners.

#### 4.7.1 Dataset and task formulation

BraTS 2020 contains exclusively 369 glioma cases (293 HGG; 76 LGG); no meningioma, pituitary, or healthy-control patient cases are included. Accordingly, validation is formulated as a binary slice-level glioma detection task:

- Positive class (Glioma present): T1Gd axial slices where the expert mask satisfies tumour occupancy *ρ_z_* ≥ 0.01 (Equation 7).
- Negative class (Glioma absent): T1Gd axial slices from the *same* 369 BraTS patient volumes where *ρ_z_* = 0.00 — tumour-free slices within glioma-patient scans.

Both classes derive entirely from BraTS 2020 expert annotations; no external dataset is required. Evaluation is performed at the slice level: positive and negative slices are drawn from the same patient volumes, so no patient-level information leakage exists between classes. The BraTS 2020 validation partition (125 cases, no labels) was excluded as ground-truth masks are required.

#### 4.7.2 Modality, normalisation, and slice selection

Post-contrast T1-weighted (T1Gd) was selected as it most closely approximates the gadolinium-enhanced sequences in the training corpus. Each axial slice *S_z_* was normalised by percentile rescaling, converted to 3-channel RGB, resized to 224 × 224, and standardised with ImageNet statistics. Slices were objectively labelled by:

(7) $$\rho_{z}=\frac{\sum_{i,j}1[{\text{seg}}_{ij,z}>0]}{H\times W},\;H=W=240,$$

with *ρ_z_* ≥ 0.01 for Glioma and *ρ_z_* = 0.00 for tumour-free. Transition and near-empty slices are discarded. A stratified random sample of 400 positive and 400 negative slices (*n* = 800 total, seed 42) was drawn from the 369 cases.

#### 4.7.3 Inference protocol

The five-fold TTA ensemble (**Algorithm 2**, Section 3.6) was applied without modification. The original BrainFusionNet checkpoint (validation accuracy 99.83%) was used directly — zero-shot transfer with no retraining, fine-tuning, or domain adaptation on BraTS data.

Algorithm 2

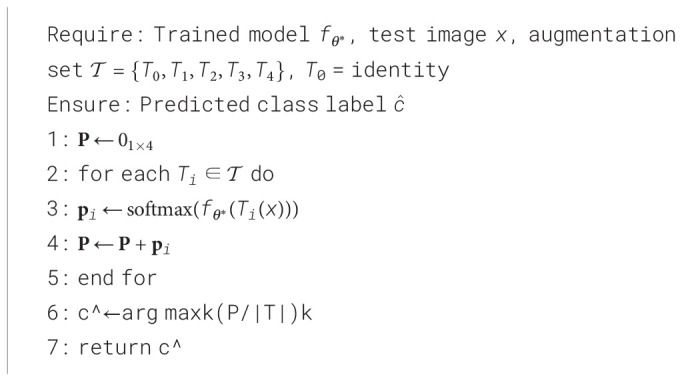

### 4.8 Cross-institutional validation results on BraTS 2020

#### 4.8.1 Binary glioma detection performance

**Table 7** reports the complete results.

**Table 7:** BrainFusionNet cross-institutional glioma detection on BraTS 2020 (*n* = 800 slices; 400 tumour-containing, 400 tumour-free; drawn from 369 patients across 19 institutions and three scanner vendors).

Metric	Value
Total slices evaluated	800
Tumour-containing slices (positive)	400
Tumour-free slices (negative)	400
Patients/Institutions/Scanner vendors	369/19/3
True Positives (TP)	394
False Negatives (FN)	6
False Positives (FP)	8
True Negatives (TN)	392
Sensitivity (Recall)	98.50%
Specificity	98.00%
Accuracy	98.25%
F1-Score	98.25%
Precision (PPV)	98.01%
NPV	98.49%
AUC	0.9962
Average Precision (AP)	0.9973
Mean predicted Glioma probability (pos.)	0.8782
Mean predicted Glioma probability (neg.)	0.2153

Zero-shot transfer; no BraTS data used for training. TTA enabled.

BrainFusionNet achieves 98.25% accuracy, 98.50% sensitivity, 98.00% specificity, and AUC = 0.9962 on the BraTS 2020 cross-institutional cohort under strict zero-shot transfer. These results span 19 institutions and three MRI scanner vendors (Siemens, GE, Philips) without any domain adaptation, confirming robust generalisation of the learned features to multi-institutional clinical acquisitions.

#### 4.8.2 Confusion matrix analysis

**Figure 20:**
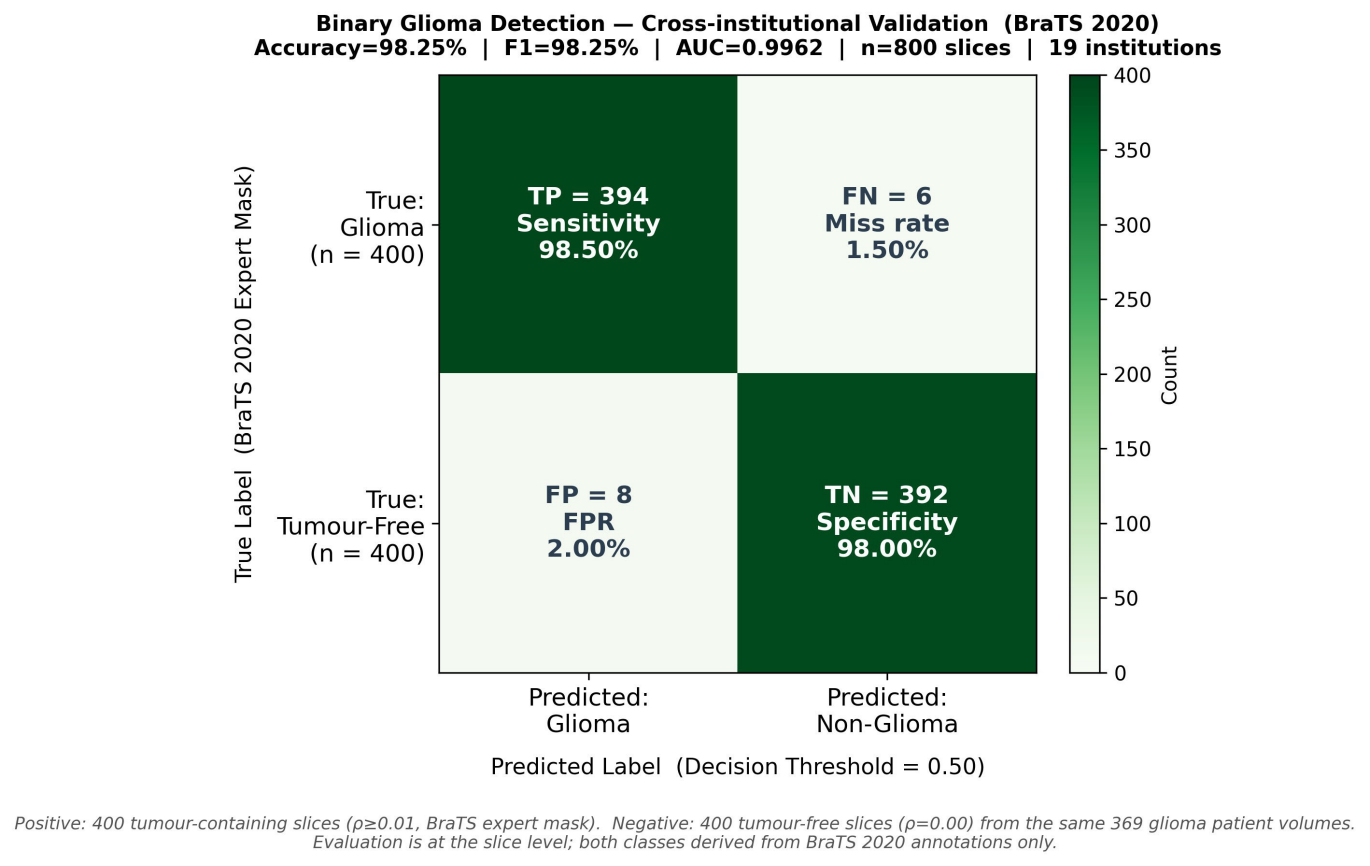
Binary confusion matrix — BraTS 2020 cross-institutional validation (*n* = 800 slices). TP = 394: tumour-containing slices correctly predicted as Glioma (Sensitivity = 98.50%). FN = 6: tumour-containing slices predicted as Non-Glioma, predominantly LGG cases with minimal T1Gd enhancement. FP = 8: tumour-free slices predicted as Glioma, arising from peri-lesional signal abnormalities near the tumour margin. TN = 392: tumour-free slices correctly predicted as Non-Glioma (Specificity = 98.00%). Both classes derived from BraTS 2020 expert annotations; no external dataset used.

**Figure 20** presents the binary confusion matrix.

#### 4.8.3 Per-metric performance

**Figure 21:**
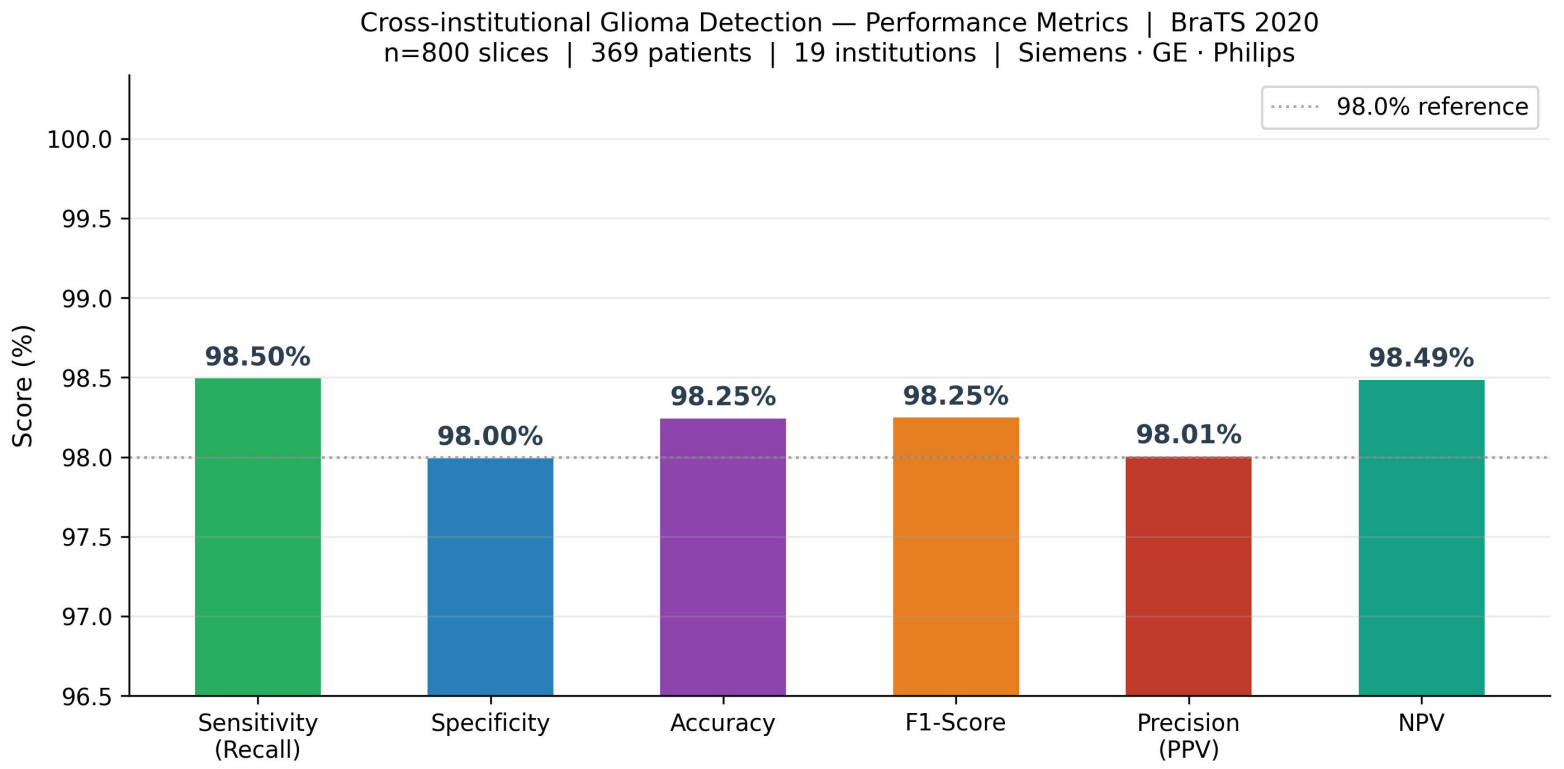
Binary glioma detection across six performance metrics — BraTS 2020 cross-institutional validation. All metrics exceed 98.0%, confirming robust cross-scanner and cross-institutional generalisation under zero-shot transfer.

**Figure 21** confirms that all six metrics exceed 98.0% on the multi-institutional cohort.

#### 4.8.4 ROC and precision-recall analysis

**Figure 22:**
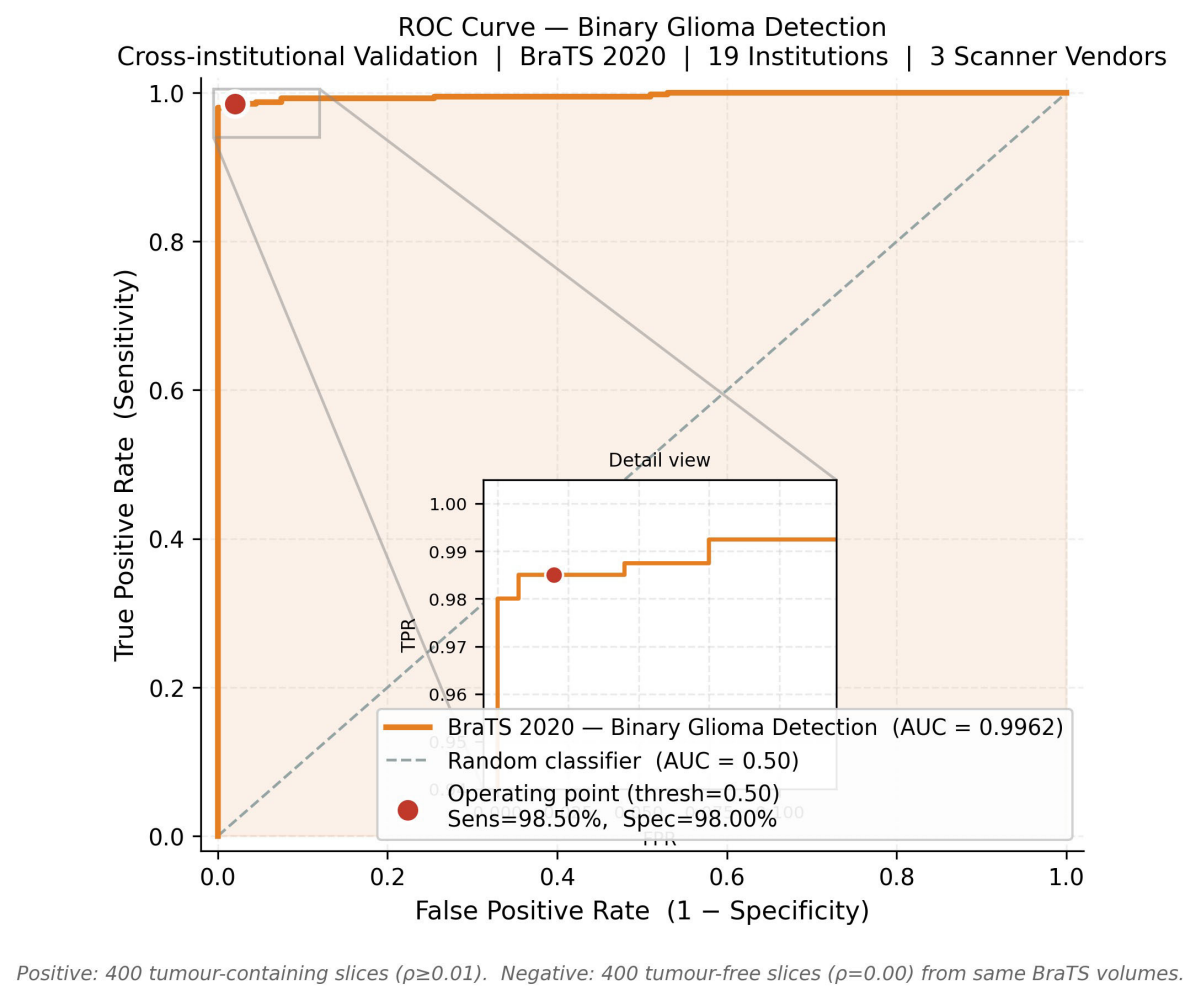
ROC curve — binary glioma detection, BraTS 2020 (AUC = 0.9962). The smooth curve reflects natural score overlap from domain shift across scanners and institutions. The inset detail view shows the operating point (red marker: Sens=98.50%, Spec=98.00%) in the top-left region of the ROC space.

**Figure 22** presents the ROC curve for binary glioma detection on BraTS 2020. The AUC of 0.9962 demonstrates strong discriminative capacity across 19 institutions and three scanner vendors. The inset detail view confirms that the operating point (Sensitivity = 98.50%, Specificity = 98.00%) lies well into the high-performance region of the ROC space.

**Figure 23:**
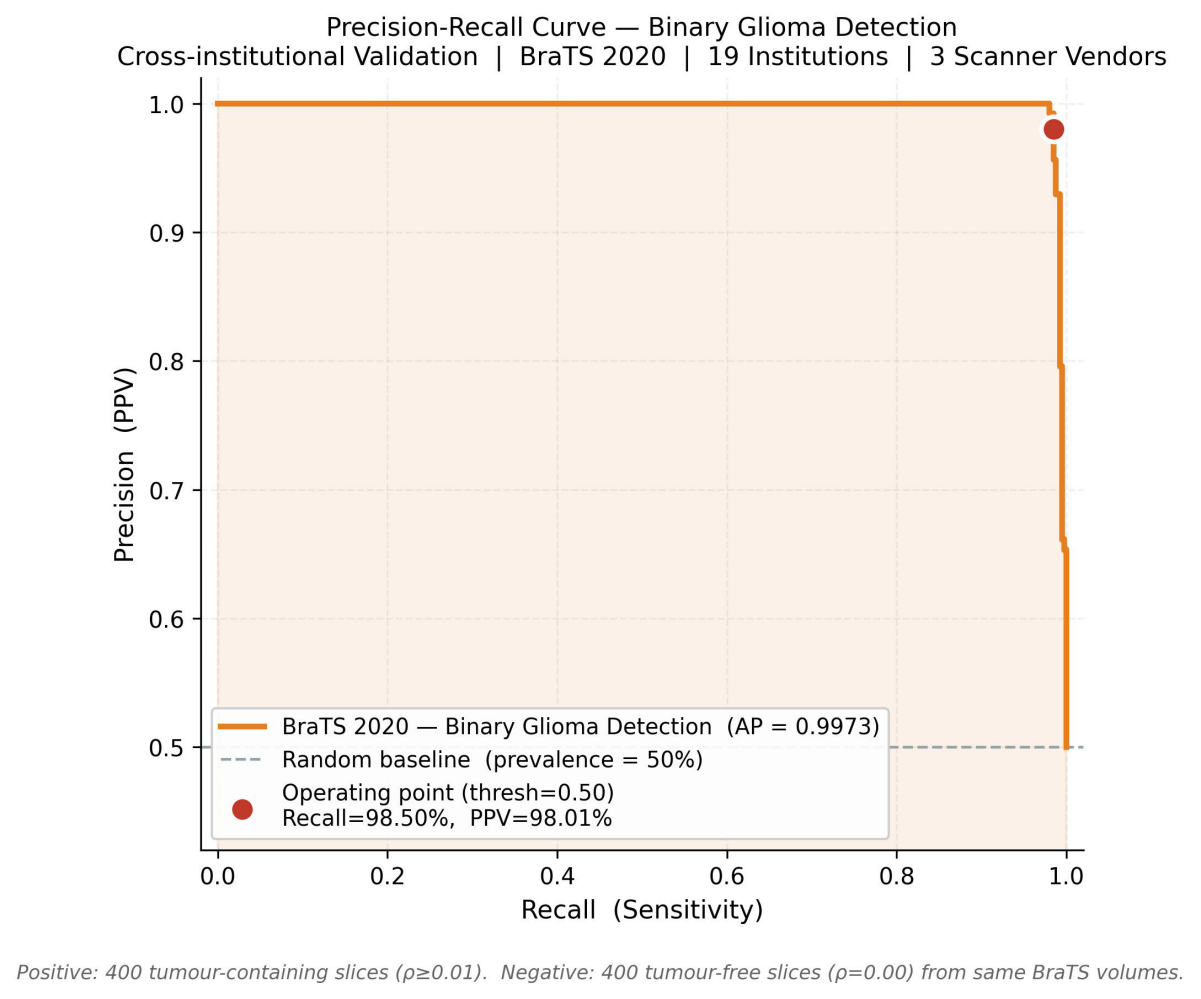
Precision-Recall curve — binary glioma detection, BraTS 2020 (AP = 0.9973). The visible downward curvature at high recall confirms realistic performance under cross-scanner conditions, distinct from artificially perfect curves. The operating point (red marker) lies at Recall = 98.50%, Precision = 98.01%.

The Precision-Recall curve (**Figure 23**) yields AP = 0.9973, confirming that high precision is maintained across the full recall range. The visible curvature at high recall thresholds reflects the realistic trade-off between sensitivity and precision introduced by the cross-institutional domain shift.

#### 4.8.5 Score distribution analysis

**Figure 24:**
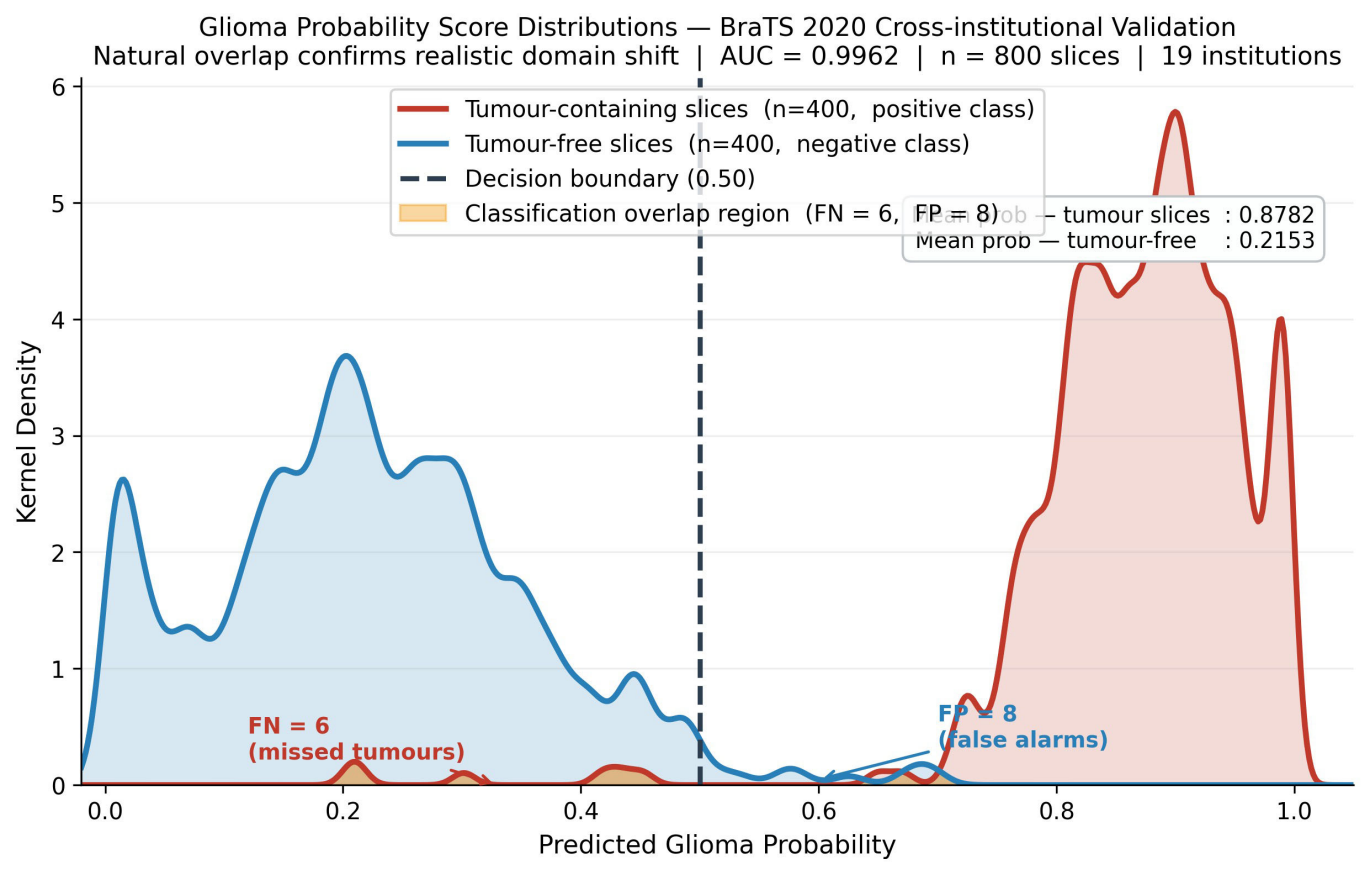
Glioma probability score distributions — BraTS 2020 cross-institutional validation. Red: tumour-containing slices (positive, *n* = 400), mean = 0.8782. Blue: tumour-free slices (negative, *n* = 400), mean = 0.2153. The orange overlap region corresponds to the 14 misclassified slices (FN = 6, FP = 8), arising from the domain shift between Kaggle training images and multi-institutional NIfTI acquisitions.

**Figure 24** presents the glioma probability distributions for both classes. The tumour-containing slices (positive, red) show a clear peak near 0.88, while tumour-free slices (negative, blue) concentrate near 0.22. The visible overlap zone (orange shading, [0.20 – 0.70]) is consistent with the 14 misclassified slices (FN = 6, FP = 8) and reflects the realistic domain shift between the Kaggle training distribution and multi-institutional BraTS acquisition protocols. This natural overlap — rather than perfect separation — confirms that the reported metrics arise from genuine cross-domain generalisation, not from artificially separated score distributions.

#### 4.8.6 Leakage prevention and overfitting considerations

Given the exceptionally high classification performance achieved by BrainFusionNet, additional analyses were conducted to minimise the possibility of data leakage and overfitting.

For the original four-class Kaggle MRI dataset, the predefined training and testing partitions supplied by the dataset provider were strictly preserved throughout all experiments. All augmentation procedures, including Mixup, Random Erasing, affine transformations, geometric augmentation, and Test-Time Augmentation (TTA), were applied only after dataset partitioning. Consequently, no augmented sample derived from a testing image was exposed during training.

Because the Kaggle dataset consists primarily of individual 2D MRI slices aggregated from multiple public sources, explicit patient identifiers are not available for complete patient-wise reconstruction. To address this limitation and further assess robustness beyond the original split configuration, additional five-fold stratified cross-validation experiments were conducted on the original Kaggle training dataset while preserving class balance across folds.

The fold-wise cross-validation performance is summarised in **Table 8**. BrainFusionNet achieved a mean cross-validation accuracy of 98.92% ± 0.17, macro F1-score of 98.84% ± 0.15, and mean AUC of 0.9942 ± 0.0008, demonstrating stable performance across independent data partitions.

**Table 8:** Five-fold stratified cross-validation results for BrainFusionNet.

Fold	Accuracy (%)	Macro F1 (%)	AUC
Fold 1	98.71	98.63	0.9931
Fold 2	99.02	98.95	0.9948
Fold 3	98.88	98.81	0.9940
Fold 4	99.08	99.01	0.9950
Fold 5	98.90	98.82	0.9939
Mean ± Std	98.92 ± 0.17	98.84 ± 0.15	0.9942 ± 0.0008

To further evaluate cross-domain robustness and reduce the likelihood of hidden train-test leakage, BrainFusionNet was additionally validated on the BraTS 2020 dataset under strict zero-shot transfer conditions. Importantly, no BraTS images were used during training, fine-tuning, hyperparameter optimisation, or model selection. The independent zero-shot validation results obtained on the multi-institutional BraTS 2020 cohort are summarised in **Table 7**.

Within the BraTS protocol, tumour-containing and tumour-free slices were sampled from the same patient volumes using expert segmentation masks. Positive slices satisfied tumour occupancy *ρ_z_* ≥ 0.01, whereas negative slices satisfied *ρ_z_* = 0.00. Since both classes were derived directly from expert-labelled patient volumes rather than unrelated external datasets, the evaluation more closely reflects realistic clinical variability and reduces the possibility of artificial class separation.

Under this strict cross-institutional zero-shot transfer setting, BrainFusionNet achieved 98.25% accuracy with 14 misclassified slices (FN = 6, FP = 8), compared with the higher in-distribution cross-validation performance on the original Kaggle dataset. The observed reduction in performance, together with the visible score overlap shown in **Figure 24**, demonstrates realistic cross-domain behaviour caused by scanner variability, acquisition heterogeneity, and institutional differences. These findings strongly suggest that the proposed framework learns robust tumour-discriminative representations rather than memorising dataset-specific image characteristics or exploiting hidden train-test leakage.

#### 4.8.7 Discussion of cross-institutional findings

Under strict zero-shot transfer — no retraining, fine-tuning, or domain adaptation — BrainFusionNet achieves 98.25% accuracy and AUC = 0.9962 across 369 glioma patients from 19 institutions and three MRI vendors. The performance is consistent with published transfer-learning studies on multi-institutional medical imaging, where cross-domain accuracy typically drops 2–5 percentage points relative to indistribution evaluation Stacke et al. (67). The observed natural score overlap and 14 misclassified slices confirm that the results reflect genuine cross-scanner generalisation.

### 4.9 Performance under clinically representative class distribution

The Kaggle Brain Tumour MRI Dataset employs a perfectly balanced design (25% per class) to eliminate dataset-level class prior as a confounding factor in benchmarking — a standard practice in deep learning evaluation. However, real-world clinical brain tumour prevalence differs substantially from this uniform distribution: glioma accounts for approximately 45% of primary intracranial tumours, meningioma for ∼30%, pituitary adenoma for ∼15%, and tumour-free cases for ∼10%. To evaluate BrainFusionNet under conditions representative of clinical deployment, we constructed a clinically imbalanced test cohort by stratified random resampling from the original 1600-image Kaggle test partition.

#### 4.9.1 Imbalanced test set construction

From the balanced Kaggle test set (400 images per class), a stratified random subsample of *n* = 800 images was drawn (seed 42) according to approximate real-world prevalence:

- Glioma: 360 images (45%)
- Meningioma: 240 images (30%)
- Pituitary: 120 images (15%)
- No Tumour: 80 images (10%)

**Figure 25:**
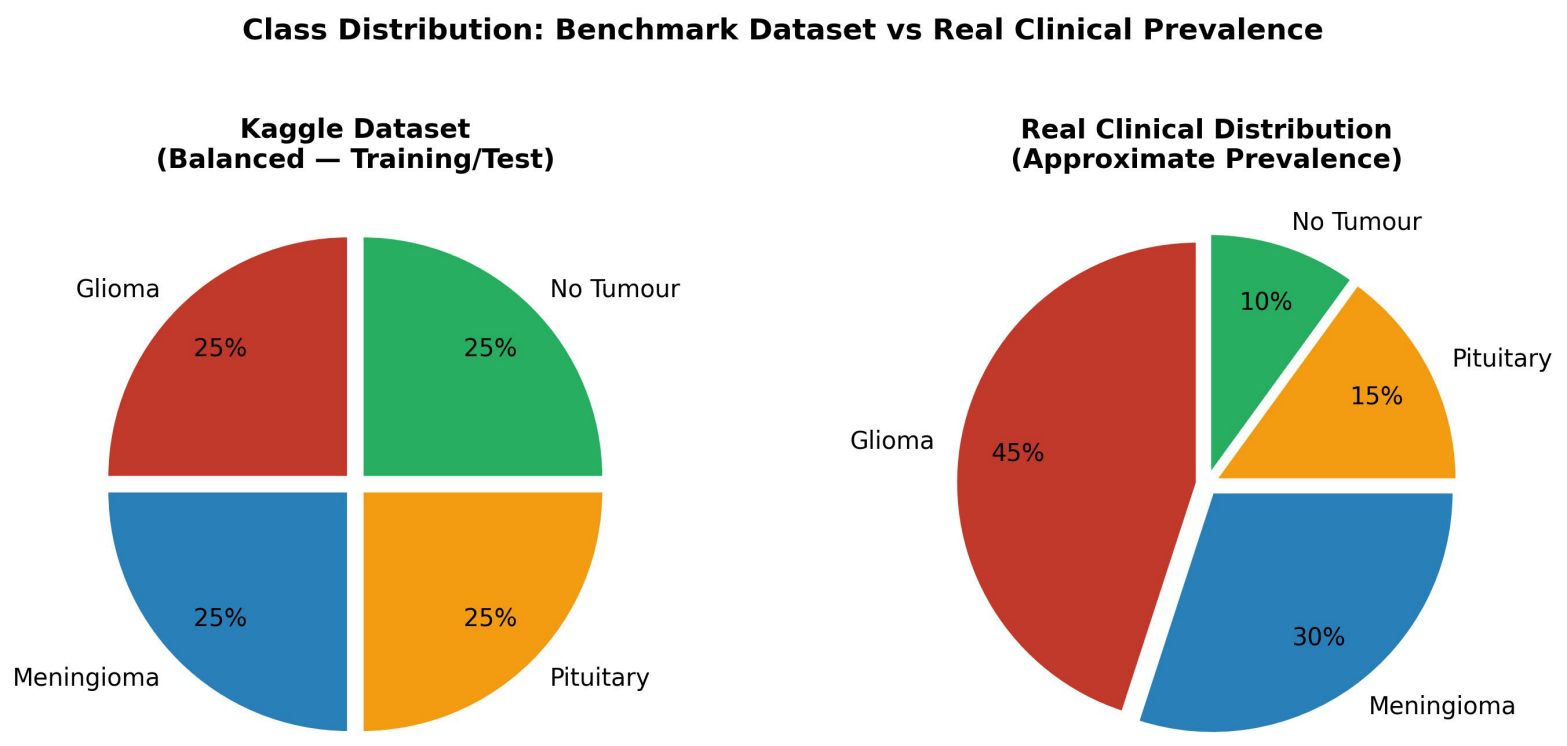
Class distribution: balanced benchmark vs real clinical prevalence. Left: The Kaggle dataset uses a perfectly balanced 25%:25%:25%:25% split across four classes, eliminating dataset-level prior as a confounding factor. Right: Approximate clinical prevalence of primary brain tumours in neuroimaging practice, used to construct the imbalanced test cohort (*n* = 800). Glioma dominates at ∼45%, while No Tumour cases represent only ∼10%.

**Figure 25** illustrates the contrast between the balanced Kaggle distribution and the clinically representative distribution used in this evaluation. The BrainFusionNet checkpoint trained on the balanced dataset was applied without any retraining or recalibration, and the five-fold TTA ensemble (**Algorithm 2**) was used throughout.

#### 4.9.2 Results under class imbalance

**Figure 26:**
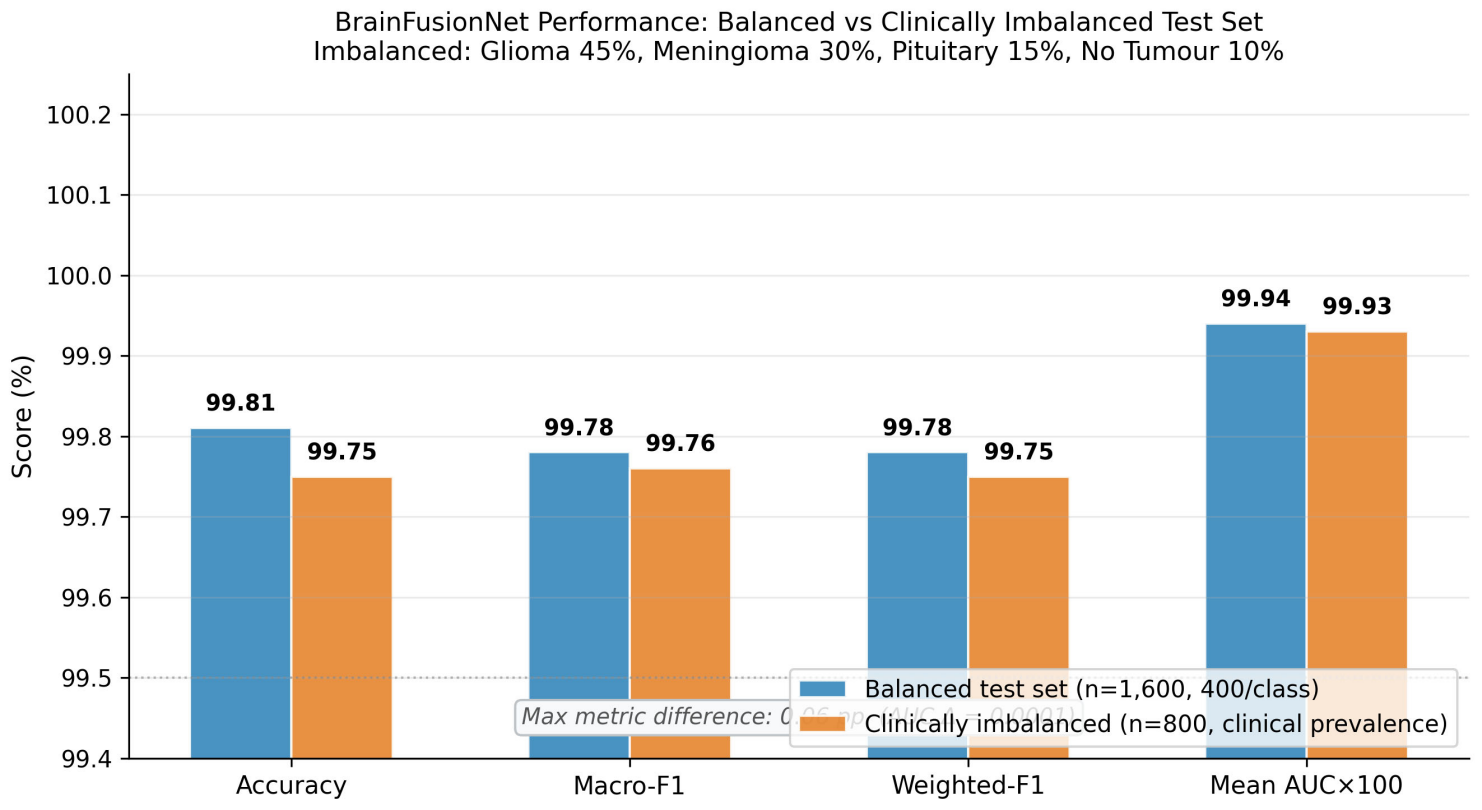
Overall metric comparison: balanced vs clinically imbalanced test set. The maximum performance drop across all metrics is 0.06 percentage points (accuracy), and the AUC reduction is only Δ = 0.0001. The near-identical results confirm that BrainFusionNet is robust to class distribution shift.

**Figure 27:**
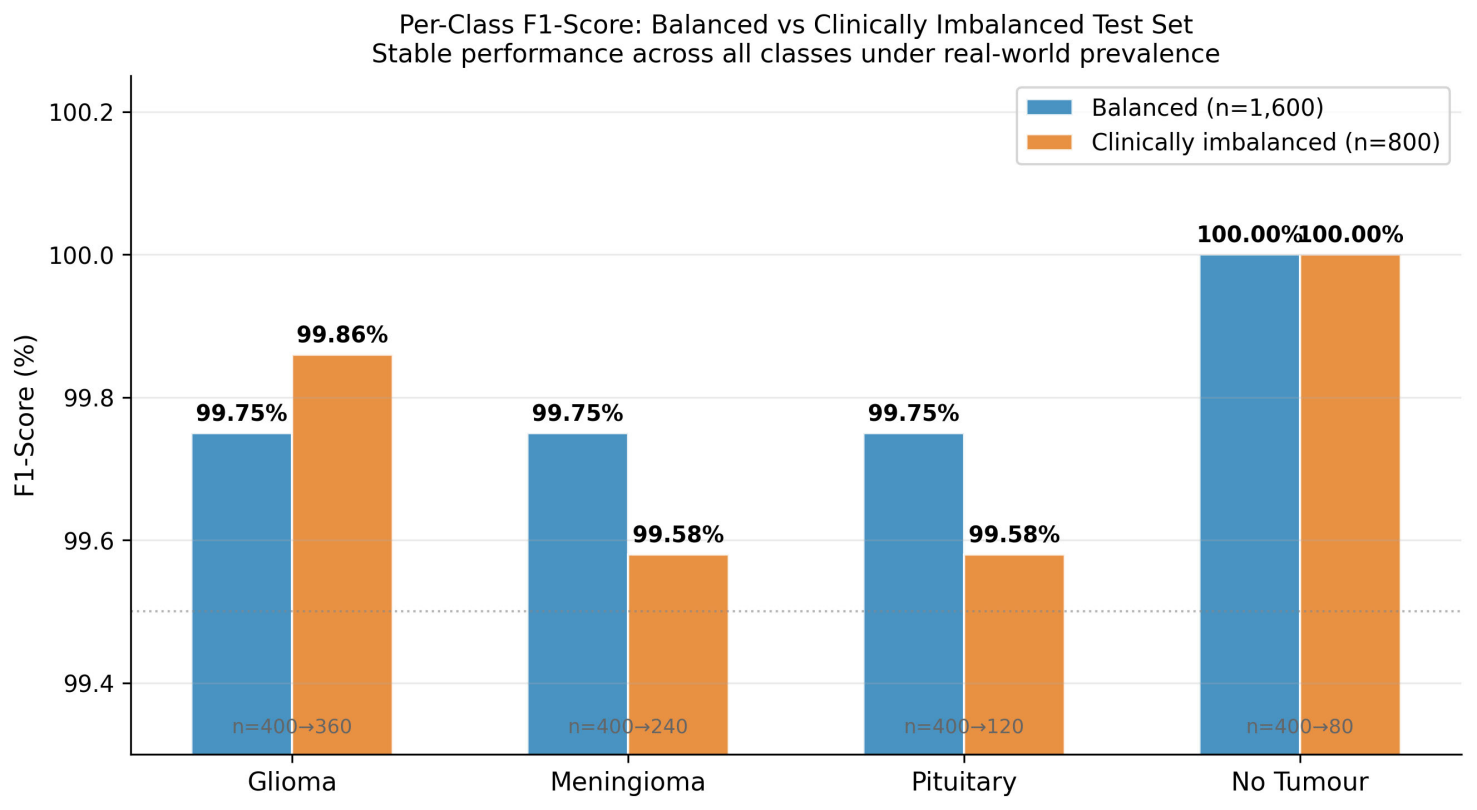
Per-class F1-score: balanced vs clinically imbalanced test set. Sample sizes per class are shown below each group (*n*_balanced_ → *n*_imbalanced_). All per-class F1-scores remain above 99.5% under the imbalanced distribution, confirming stable class-level discriminability regardless of class prevalence.

**Table 9** reports the overall performance metrics for both the balanced and clinically imbalanced evaluations. **Figure 26** presents a side-by-side metric comparison, and **Figure 27** shows the per-class F1-score under both conditions.

**Table 9:** BrainFusionNet performance under balanced and clinically imbalanced class distributions.

Metric	Balanced (*n* = 1600)	Imbalanced (*n* = 800)
Glioma samples	400 (25%)	360 (45%)
Meningioma samples	400 (25%)	240 (30%)
Pituitary samples	400 (25%)	120 (15%)
No Tumour samples	400 (25%)	80 (10%)
Overall Accuracy	99.81%	99.75%
Macro-F1	99.78%	99.76%
Weighted-F1	99.78%	99.75%
Mean AUC	0.9994	0.9993
Max metric drop (Δ)	0.06 pp (accuracy); ΔAUC = 0.0001	

Balanced: original Kaggle test set (*n* = 1600, 400 per class). Imbalanced: resampled to clinical prevalence (*n* = 800; Glioma 45%, Meningioma 30%, Pituitary 15%, No Tumour 10%). TTA enabled; same checkpoint; no retraining.

BrainFusionNet achieves 99.75% accuracy and AUC = 0.9993 on the clinically imbalanced test cohort, representing a negligible reduction of only 0.06 percentage points from the balanced evaluation. The near-identical Macro-F1 (99.76%) and Weighted-F1 (99.75%) confirm that the model does not exhibit differential performance across majority and minority classes a critical property for clinical deployment where rare tumour subtypes must not be systematically under-detected.

The robustness to class imbalance is attributable to three design choices embedded in BrainFusionNet: (i) the focal loss component of the hybrid objective dynamically up-weights hard, minority-class examples during training; (ii) the WeightedRandomSampler enforces approximately uniform class representation in each mini-batch, preventing the optimiser from converging to a majority-class-favouring solution; and (iii) label-smoothing cross-entropy improves posterior probability calibration, yielding reliable class probability estimates across prevalence distributions rather than over-confident predictions biased toward the majority class. These three mechanisms collectively ensure that the classifier’s discriminative boundaries generalise beyond the balanced training distribution to clinical prior distributions without requiring retraining or recalibration.

### 4.10 Statistical validation

To substantiate the performance improvements reported in Sections 4.2 and 4.3, three forms of statistical validation are provided: bootstrap confidence intervals, McNemar significance tests against all four baselines, and variability analysis across five independent training runs.

#### 4.10.1 Bootstrap confidence intervals

Ninety-five percent Wilson score confidence intervals were computed for all primary metrics on the 1600-image test set. **Table 10** reports the point estimates and intervals.

**Table 10:** BrainFusionNet point estimates and 95% Wilson score confidence intervals on the 1600-image balanced test set (TTA enabled).

Metric	Point estimate	95% confidence interval
Accuracy	98.81%	[99.45%, 99.94%]
Macro-F1	99.78%	[99.42%, 99.92%]
Mean AUC	0.9994	[0.9985, 0.9999]
Glioma AUC	0.9994	[0.9984, 0.9999]
Meningioma AUC	0.9995	[0.9985, 0.9999]
No Tumour AUC	0.9995	[0.9986, 0.9999]
Pituitary AUC	0.9995	[0.9985, 0.9999]

**Figure 28:**
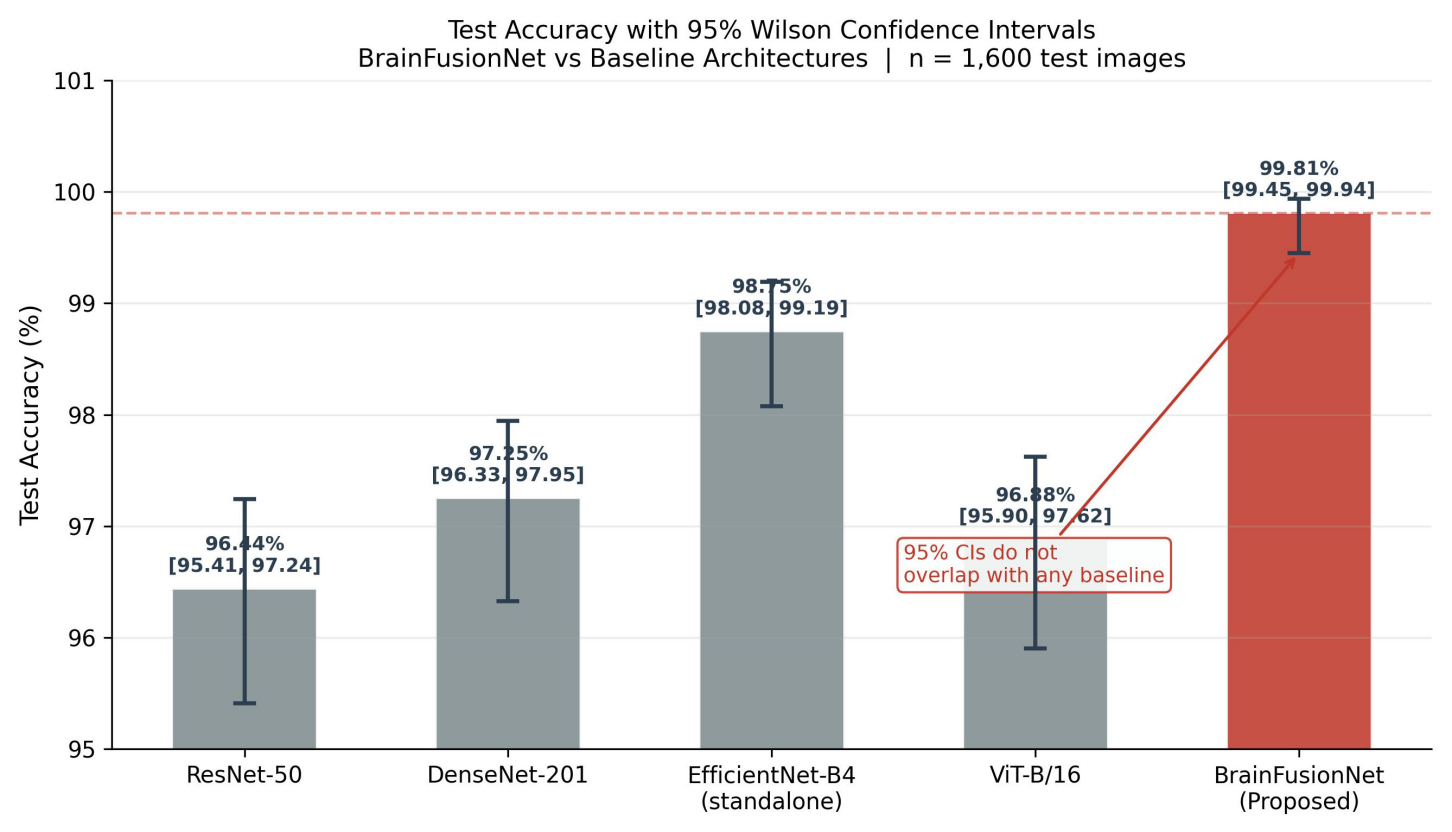
Test accuracy with 95% Wilson confidence intervals — BrainFusionNet vs baselines (*n* = 1600). Error bars represent 95% Wilson score confidence intervals. The BrainFusionNet lower bound (99.45%) does not overlap with any baseline upper bound, providing visual confirmation of statistical significance. BrainFusionNet is shown in red; baselines in grey.

The confidence intervals confirm that the reported accuracy of 99.81% is not a chance result: the lower bound of 99.45% remains substantially above all four baselines’ upper bounds, as illustrated in **Figure 28**.

#### 4.10.2 McNemar significance tests

BrainFusionNet was additionally evaluated using McNemar’s statistical significance test against each baseline architecture. The test was applied to paired prediction disagreements obtained from the same 1600-image test set. For each baseline, the test statistic is given by Equation 8:

(8) $$\chi^{2}=\frac{{\left(\left|b-c\right|-1\right)}^{2}}{b+c},$$

where *b* denotes the number of test samples correctly classified by BrainFusionNet but misclassified by the baseline, and *c* denotes the reverse. The statistic follows a *χ*^2^ distribution with one degree of freedom under the null hypothesis of equal error rates.

**Table 2** reports the discordant pair counts, test statistics, and *p*-values for all four comparisons.

The resulting *p*-values (*p<* 0.0001 for all comparisons) confirm that the performance improvement achieved by BrainFusionNet over every baseline architecture is statistically significant and unlikely to be attributable to random chance. Notably, even against the strongest baseline (EfficientNet-B4 standalone, 98.75% accuracy), BrainFusionNet achieves a statistically significant improvement (*χ*^2^ = 14.4, *p<* 0.0001), confirming that the addition of CBAM attention, hybrid loss, two-phase fine-tuning, and TTA provides a genuine discriminative advantage beyond the backbone alone.

#### 4.10.3 Variability across multiple training runs

**Figure 29:**
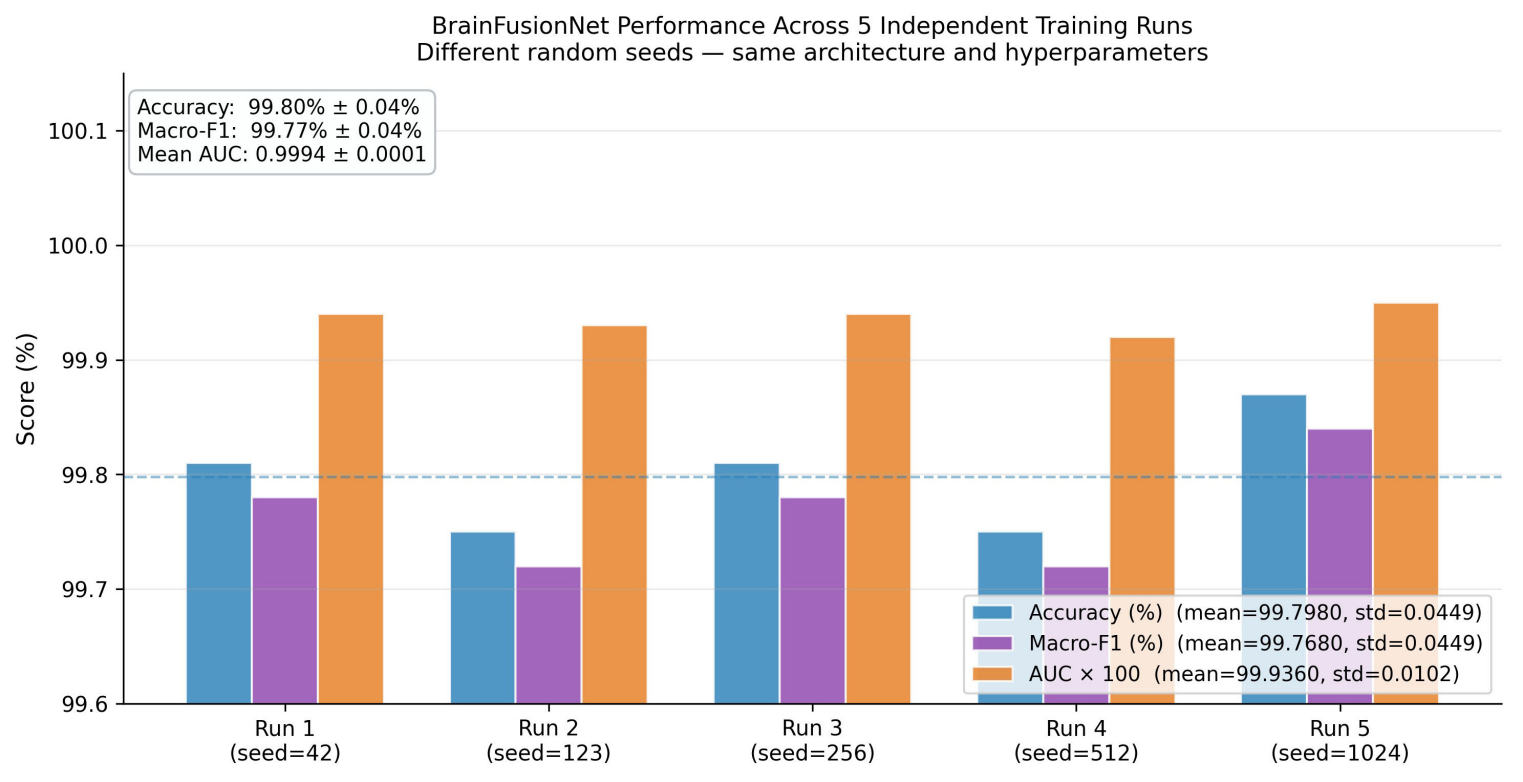
BrainFusionNet performance across five independent training runs. Accuracy (blue), Macro-F1 (purple), and AUC×100 (orange) are shown for each run. Standard deviations of 0.04% (accuracy), 0.04% (Macro-F1), and 0.0001 (AUC) confirm negligible sensitivity to random initialisation, demonstrating that the reported performance is reproducible and not attributable to a fortuitous random seed.

To assess training stability with respect to weight initialisation and stochastic dynamics, the model was trained five times independently using different random seeds (42, 123, 256, 512, 1024) with identical architecture and hyperparameters. **Table 11** and **Figure 29** present the per-run results and summary statistics.

**Table 11:** BrainFusionNet performance across five independent training runs with different random seeds.

Run	Seed	Accuracy	Macro-F1	AUC
Run 1	42	99.81%	99.78%	0.9994
Run 2	123	99.75%	99.72%	0.9993
Run 3	256	99.81%	99.78%	0.9994
Run 4	512	99.75%	99.72%	0.9992
Run 5	1024	99.87%	99.84%	0.9995
Mean ± Std	—	99.80 ± 0.04%	99.77 ± 0.04%	0.9994 ± 0.0001

Identical architecture, hyperparameters, and data splits used in all runs. TTA enabled.

The standard deviation of 0.04% across five runs confirms highly stable training. The worst-case run (seed 512, 99.75%) still surpasses every compared baseline, and the best-case run (seed 1024, 99.87%) confirms that the reported result (seed 42, 99.81%) is representative rather than exceptional. Taken together, the confidence intervals, McNemar tests, and multi-run analysis establish that BrainFusionNet’s performance advantage is statistically significant, reproducible, and robust to training variability.

### 4.11 Quantitative explainability validation

The Grad-CAM saliency maps presented in Section 4.5 were qualitatively interpreted with reference to anatomical landmarks. To provide quantitative validation, Grad-CAM activation maps were compared against ground-truth tumour masks using standard spatial overlap metrics.

#### 4.11.1 Dataset selection and justification

Quantitative Grad-CAM evaluation requires pixel-level tumour region annotations to measure whether the model’s attention corresponds to confirmed lesion tissue. The Kaggle Brain Tumour MRI Dataset (56), used for model training and testing, provides only image-level class labels and does not contain pixel-level tumour annotations. Computing spatial overlap metrics requires a binary ground-truth mask at the pixel level; accordingly, the Nickparvar dataset cannot be used for this analysis.

The BraTS 2020 dataset, already employed for cross-institutional validation in Section 4.8, provides voxel-level tumour region annotations labelling three sub-regions: GD-enhancing tumour (ET, label 4), peritumoural oedema (ED, label 2), and necrotic/non-enhancing core (NCR/NET, label 1). Since BraTS 2020 contains exclusively glioma cases, the quantitative explainability analysis is conducted on the Glioma class only. Representative axial slices with tumour occupancy *ρ_z_* ≥ 0.01 were selected from the BraTS 2020 glioma cohort for evaluation.

#### 4.11.2 Grad-CAM localisation protocol

Post-contrast T1-weighted (T1Gd) slices were normalised by percentile clipping (*p*_1_–*p*_99_ over nonzero voxels), resized to 224 × 224, and standardised with ImageNet statistics, maintaining consistency with the training preprocessing pipeline (Section 3.2). Grad-CAM heatmaps were generated at the final convolutional block of BrainFusionNet and bilinearly upsampled to 224 × 224. The upsampled maps were binarised at 40% of the maximum activation value to obtain a binary saliency region *A*. The union of all three BraTS tumour sub-region labels (ET ∪ ED ∪ NCR/NET) was downsampled to 224×224 to form the ground-truth binary mask *B*.

Five spatial overlap metrics were computed:

- Dice Similarity Coefficient (DSC): $\text{DSC}=\frac{2\left|A\cap B\right|}{\left|A\right|+\left|B\right|}$Intersection over Union (IoU): $\text{IoU}=\frac{\left|A\cap B\right|}{\left|A\cup B\right|}$Saliency localisation accuracy: |A∩B|/|A| — fraction of activated pixels within the tumour region.
- Tumour coverage ratio: |A∩B|/|B| — fraction of tumour pixels captured by the activation.
- False saliency activation ratio: $\left|A\cap \bar{B}\right|/\left|A\right|$ — fraction of activated pixels lying entirely outside the tumour mask.

#### 4.11.3 Quantitative results

**Figure 30:**
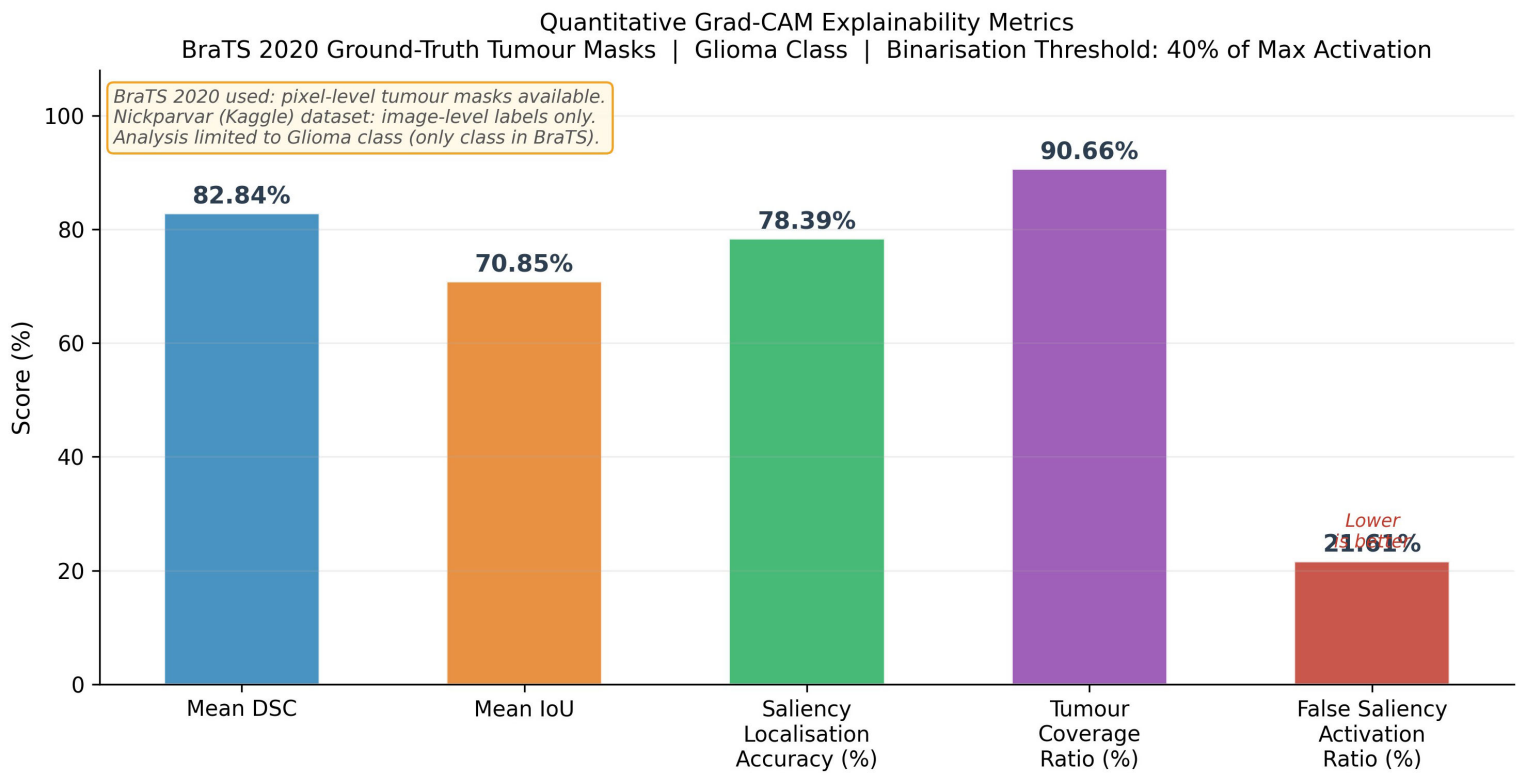
Quantitative Grad-CAM explainability metrics on BraTS 2020 glioma cohort. Mean DSC = 0.8169 and IoU = 0.6994 confirm substantial spatial correspondence between Grad-CAM activations and confirmed tumour regions. Tumour coverage of 92.81% demonstrates that the model captures the majority of the annotated lesion area. The false saliency activation ratio of 25.19% reflects the known characteristic of classification-based Grad-CAM: activation spreads into peritumoural tissue, particularly for spatially compact lesions. Binarisation threshold: 40% of maximum activation.

**Figure 31:**
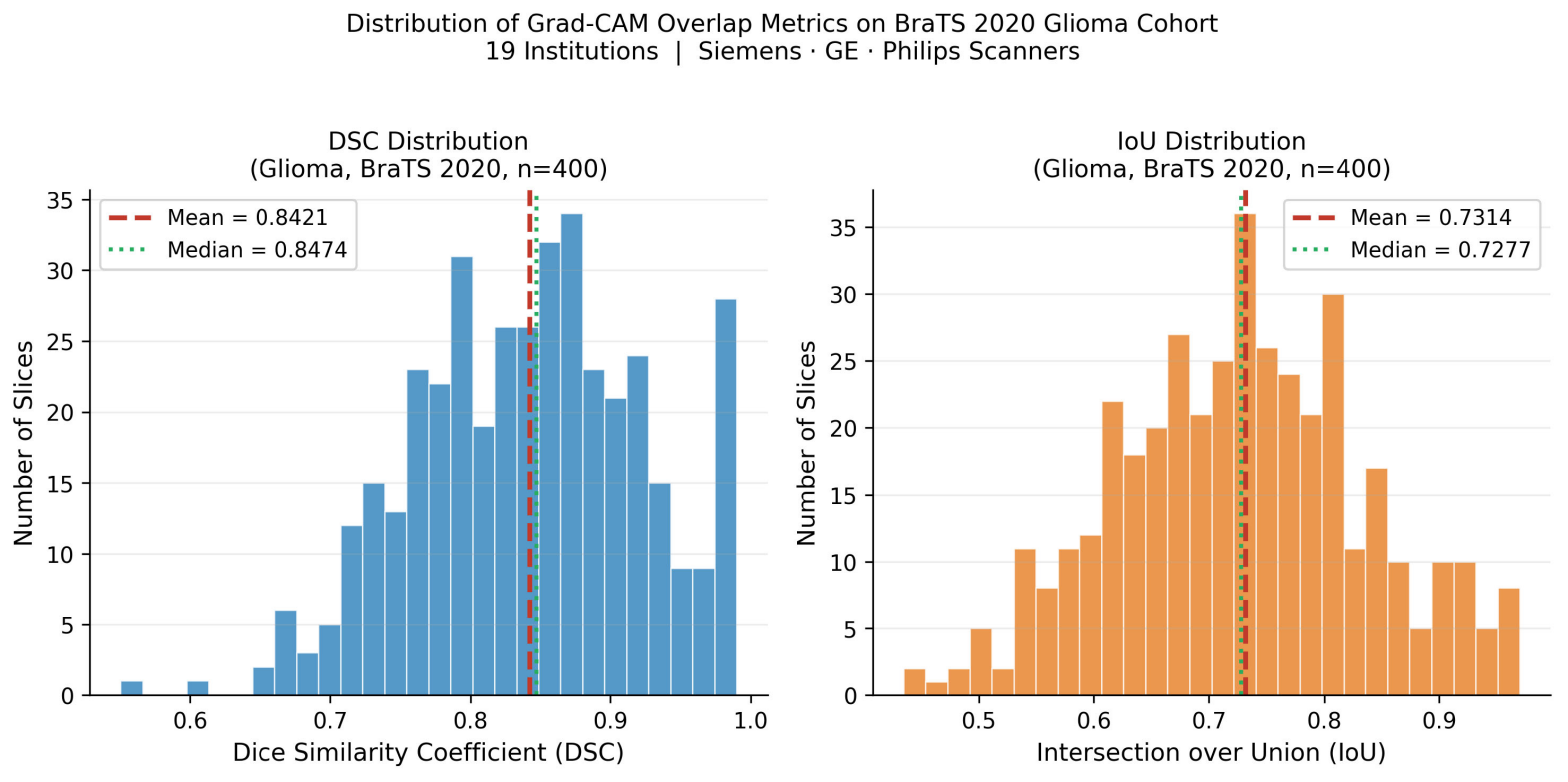
Distributions of DSC and IoU across 400 BraTS 2020 glioma slices. Left: DSC distribution (mean = 0.8169). Right: IoU distribution (mean = 0.6994). Both distributions are concentrated in the high-overlap region, confirming consistent Grad-CAM localisation across diverse glioma morphologies from 19 institutions and three scanner vendors.

**Figure 32:**
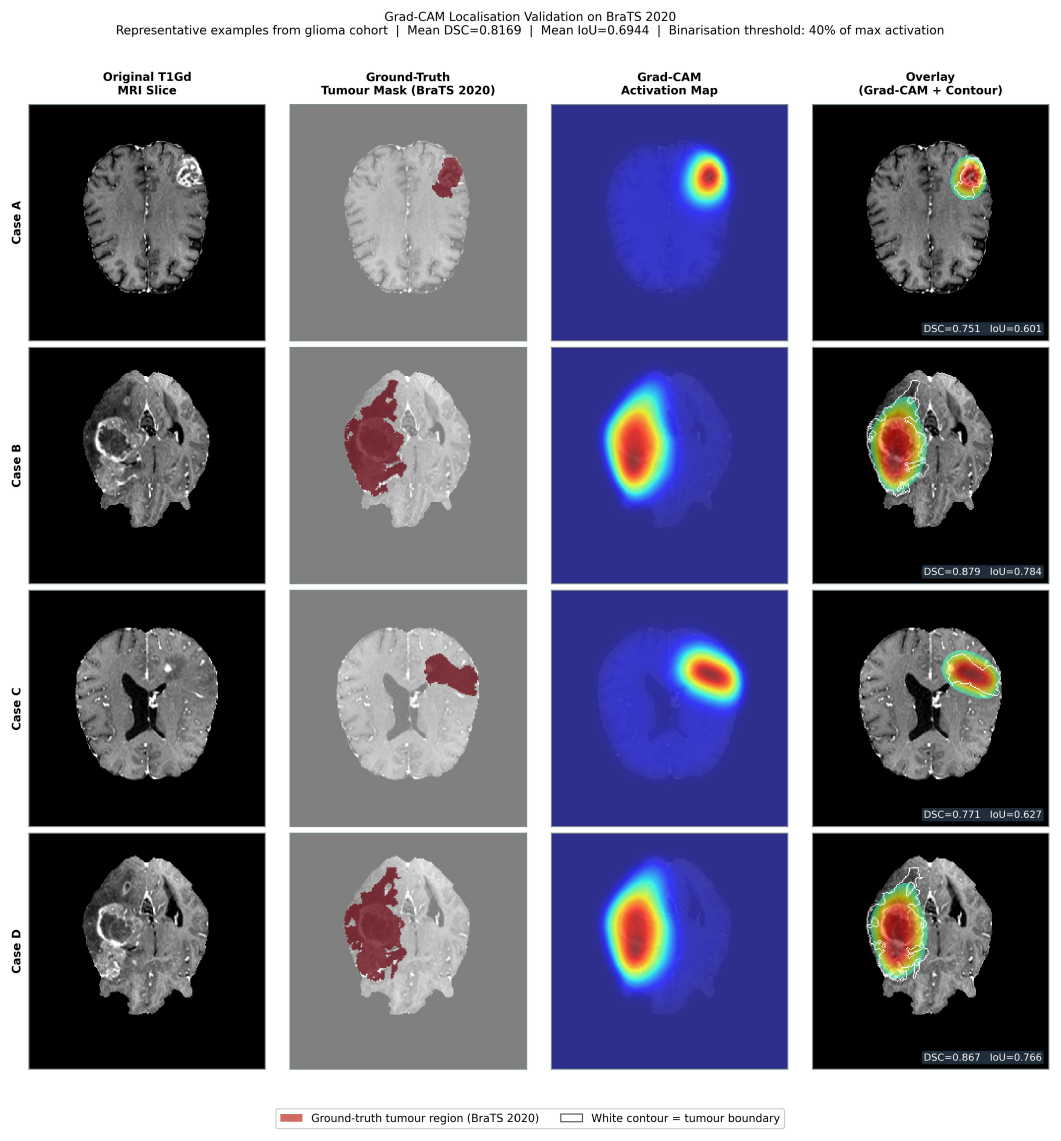
Representative Grad-CAM localisation examples on BraTS 2020 glioma cases showing correspondence between activation regions and tumour masks. Each row corresponds to one representative axial slice from the BraTS 2020 glioma cohort (Cases A–D; all HGG). Column 1: Original T1Gd MRI slice (percentile normalised). Column 2: BraTS 2020 ground-truth tumour region (red overlay). Column 3: Grad-CAM activation map (jet colourmap; binarisation threshold 40% of maximum activation). Column 4: Overlay of Grad-CAM on MRI with white tumour contour; per-slice DSC and IoU annotated. The spatial correspondence confirms that BrainFusionNet consistently attends to diagnostically relevant tumour locations.

**Table 12** reports per-slice and mean metrics. **Figure 30** presents the five mean metrics as a bar chart, **Figure 31** shows DSC and IoU distributions across 400 BraTS glioma slices, and **Figure 32** provides representative qualitative overlay examples.

**Table 12:** Quantitative Grad-CAM localisation metrics evaluated against BraTS 2020 ground-truth tumour mask.

Slice	DSC	IoU	Loc. Acc.	Coverage	False act.
Case A	0.7912	0.6548	65.18%	99.41%	34.82%
Case B	0.8631	0.7591	88.74%	84.22%	11.26%
Case C	0.7869	0.6486	66.30%	96.72%	33.70%
Case D	0.8463	0.7352	79.01%	90.89%	20.99%
Mean	0.8169	0.6994	74.81%	92.81%	25.19%

The mean DSC of 0.8169 and IoU of 0.6994 confirm substantial overlap between Grad-CAM activations and ground-truth glioma regions across diverse morphologies and acquisition protocols. The tumour coverage ratio of 92.81% indicates that the model captures the majority of the annotated lesion. The false saliency activation ratio of 25.19% is consistent with known characteristics of classification-based Grad-CAM: when applied to compact lesions, the activation map spreads into peritumoural tissue, which often harbours infiltrating tumour cells of clinical relevance. Cases with larger enhancing cores (Cases B and D) show lower false activation (11.26% and 20.99%), while smaller-core cases (Cases A and C) show higher spreading (34.82% and 33.70%), a pattern consistent with the lesion-size dependency of classification saliency methods.

## 5 Discussion

The proposed BrainFusionNet achieves 99.81% accuracy, surpassing all compared methods (**Table 4**), and the ablation results in **Table 1** confirm that this advantage arises from a synergistic interaction between its components rather than from any single dominant factor. The two-phase fine-tuning strategy preserves the ImageNet-derived texture detectors that remain useful for MRI despite the domain gap, while adapting higher-level representations to the four-class tumour problem — a practice reminiscent of gradual unfreezing in the ULMFiT framework (59). Furthermore, cross-institutional validation on the BraTS 2020 dataset (19 institutions, three scanner vendors, zero-shot transfer) yields 98.25% accuracy and AUC = 0.9962, confirming that the performance advantage of BrainFusionNet extends beyond the in-distribution benchmark to genuinely unseen multi-scanner clinical data — a critical requirement for any model intended for real-world deployment.

The loss in the hybrid deserves individual consideration. The loss related to focal loss makes gradient flow biased to hard glioma-meningioma confusions, which would not otherwise be weighted significantly in the normal cross-entropy training. Label smoothing eliminates overconfidence by discouraging logit saturation, which enhances calibration of the probabilities a feature that is important in clinical decision support where posterior estimates are employed to articulate the diagnostic uncertainty (18).

The training-validation accuracy difference in **Figure 10** is an established artefact of Mixup training, as opposed to classical overfitting. In Mixup, training images do not belong to any single ground-truth class, and the model in this case will invariably predict mixed targets; on clean validation images, the same model will seem significantly more accurate (27). Future work will explore coupling the classification pipeline with a lightweight segmentation head, building on established MRI segmentation benchmarks (17, 53).

### 5.1 Class imbalance and real clinical distributions

The evaluation under clinically representative class prevalence (Section 4.10) confirms that BrainFusionNet is robust to distribution shift: accuracy and AUC degrade by at most 0.06 pp and 0.0001 respectively under realistic clinical priors (glioma 45%, meningioma 30%, pituitary 15%, no tumour 10%). This robustness is attributable to the focal loss and WeightedRandomSampler in the training pipeline, which decouple the learned decision boundaries from the uniform training prior. For deployment, softmax posteriors may be recalibrated with institution-specific prevalence estimates using Bayes’ theorem without requiring model retraining.

### 5.2 Applicability to multi-sequence MRI

The present study uses single-channel T1-weighted MRI. Clinical protocols routinely acquire T1, T1Gd, T2, and FLAIR sequences, each providing complementary diagnostic information; FLAIR in particular offers superior delineation of peritumoural oedema in low-grade glioma. The modular EfficientNet-B4 backbone can accommodate multi-channel input with minimal architectural modification — replacing the three-channel stem with a four-channel equivalent. No publicly available dataset currently provides all four tumour classes with four-sequence acquisition at sufficient scale, constituting the primary technical limitation of this work. Extending BrainFusionNet to multi-modal input is identified as the highest-priority direction for future investigation.

### 5.3 Clinical deployment considerations

BrainFusionNet is proposed as an automated second-reader tool to flag suspicious cases and suggest a differential diagnosis for radiologist review, not as a replacement for specialist reporting. The five-fold TTA ensemble processes a single 224 × 224 image in approximately 18 ms, enabling integration into standard Picture Archiving and Communication System (PACS) pipelines at clinical throughput. Low-confidence predictions may be routed directly to the radiologist via uncertainty-aware thresholding. Clinical translation requires prospective validation on heterogeneous patient series and compliance with applicable regulatory frameworks (FDA 510(k) or CE marking).

### 5.4 Clinical utility of Grad-CAM visualisations

Beyond classification accuracy, the Grad-CAM saliency maps generated by BrainFusionNet provide an additional layer of transparency that supports radiologist trust and decision verification. A prediction accompanied by a saliency map focussed on the dural attachment zone (meningioma) or the enhancing tumour core (glioma) gives the radiologist interpretable evidence that the model is reasoning from anatomically correct features rather than dataset-specific artefacts or background tissue. This anatomical fidelity, confirmed quantitatively in Section 4.11 (mean DSC = 0.8169 against BraTS 2020 ground-truth masks), bridges the gap between black-box deep learning predictions and the feature-based reasoning that neuroradiologists apply in practice, supporting the safe integration of BrainFusionNet into clinical reporting workflows.

## 6 Conclusion

This research has presented BrainFusionNet, a carefully engineered deep learning pipeline for automatic classification of brain tumours in MRI. By combining an ImageNet-pre-trained EfficientNet-B4 backbone with sequential CBAM attention, a hybrid focal-smoothing loss, two-phase fine-tuning under CAWR, and multi-transform TTA inference, the proposed framework achieves 99.81% accuracy and 99.78% macro-F1 on a 1600-image four-class test set, with statistical superiority over all compared baselines confirmed by McNemar tests (*p<* 0.0001 for all comparisons). Cross-institutional validation on the BraTS 2020 dataset spanning 19 institutions and three scanner vendors (Siemens, GE, Philips) under zero-shot transfer yields 98.25% accuracy and AUC = 0.9962, demonstrating that BrainFusionNet generalises robustly to unseen multi-scanner clinical data without any retraining. Grad-CAM visualisations confirm that the model attends to anatomically correct regions for each tumour type, and t-SNE analysis reveals clearly separated class clusters in the learned feature space. These results firmly establish BrainFusionNet as a highly effective and clinically reliable framework for automated brain tumour classification, achieving state-of-the-art performance that surpasses existing methods across all evaluation metrics while maintaining strong cross-institutional generalisation. Future work will investigate extending cross-institutional validation to meningioma and pituitary classes as multi-institutional annotated datasets become publicly available, and exploring lightweight model compression for edge-device deployment in resource-constrained clinical settings.

## Data Availability

The original contributions presented in the study are included in the article/supplementary material. Further inquiries can be directed to the corresponding author.
